# Antitrypanosomal and Antileishmanial Activity of Chalcones and Flavanones from *Polygonum salicifolium*

**DOI:** 10.3390/pathogens10020175

**Published:** 2021-02-05

**Authors:** Ahmed M. Zheoat, Samya Alenezi, Ehab Kotb Elmahallawy, Marzuq A. Ungogo, Ali H. Alghamdi, David G. Watson, John O. Igoli, Alexander I. Gray, Harry P. de Koning, Valerie A. Ferro

**Affiliations:** 1 Strathclyde Institute of Pharmacy and Biomedical Sciences, University of Strathclyde, Glasgow G4 0RE, UK; ahmedpharm.2013@gmail.com (A.M.Z.); s-alenezi@strath.ac.uk (S.A.); D.G.Watson@strath.ac.uk (D.G.W.); igolij@gmail.com (J.O.I.); a.i.gray345@gmail.com (A.I.G.); v.a.ferro@strath.ac.uk (V.A.F.); 2 Al-Manara College for Medical Sciences, Misan 10028, Iraq; 3 Institute of Infection, Immunity and Inflammation, College of Medical, Veterinary and Life Sciences, University of Glasgow, Glasgow G12 8TA, UK; eehaa@unileon.es (E.K.E.); 2226184U@student.gla.ac.uk (M.A.U.); Ayfan24@hotmail.com (A.H.A.); 4 Department of Zoonoses, Faculty of Veterinary Medicine, Sohag University, Sohag 82524, Egypt; 5 Department of Veterinary Pharmacology and Toxicology, Faculty of Veterinary Medicine, Ahmadu Bello University, Zaria 810107, Nigeria; 6 Biology Department, Faculty of Science, Albaha University, Albaha 7738-65799, Saudi Arabia; 7 Phytochemistry Research Group, Department of Chemistry, University of Agriculture, Makurdi 2373, Nigeria

**Keywords:** *Polygonum salicifolium*, chalcone, flavanone, *Leishmania mexicana*, *Trypanosoma brucei brucei*, *Trypanosoma congolense*

## Abstract

Trypanosomiasis and leishmaniasis are a group of neglected parasitic diseases caused by several species of parasites belonging to the family Trypansomatida. The present study investigated the antitrypanosomal and antileishmanial activity of chalcones and flavanones from *Polygonum salicifolium*, which grows in the wetlands of Iraq. The phytochemical evaluation of the plant yielded two chalcones, 2′,4′-dimethoxy-6′-hydroxychalcone and 2′,5′-dimethoxy-4′,6′-dihydroxychalcone, and two flavanones, 5,7-dimethoxyflavanone and 5,8-dimethoxy-7-hydroxyflavanone. The chalcones showed a good antitrypanosomal and antileishmanial activity while the flavanones were inactive. The EC_50_ values for 2′,4′-dimethoxy-6′-hydroxychalcone against *Trypanosoma brucei brucei* (0.5 μg/mL), *T. congolense* (2.5 μg/mL), and *Leishmania mexicana* (5.2 μg/mL) indicated it was the most active of the compounds. None of the compounds displayed any toxicity against a human cell line, even at 100 µg/mL, or cross-resistance with first line clinical trypanocides, such as diamidines and melaminophenyl arsenicals. Taken together, our study provides significant data in relation to the activity of chalcones and flavanones from *P. salicifolium* against both parasites in vitro. Further future research is suggested in order to investigate the mode of action of the extracted chalcones against the parasites.

## 1. Introduction

The marshes of Iraq are considered to be the largest ecosystem in the Middle East and Western Eurasia [1]. More than one hundred species of aquatic and amphibious plants have been recorded in this area. Ancient Mesopotamians used some of the plants for a range of medicinal and culinary purposes. In modern Mesopotamia, Marsh Arabs also use plants from the marshes for medicinal and healing purposes [1]. *Polygonum salicifolium* is a common species found in the wetlands, and it is an important source of food in the localities [2]. There is little information on the phytochemical constituents and the potential biological activities of this plant. Previous studies on *P. salicifolium* indicated flavonoid glycosides [3] and flavonol glycosides [4] as predominant constituents in the aerial parts of the plant. These classes of compounds are well known for their antioxidant effects [4]. Midiwo et al. [5] described the wide use of Polygonaceae as ethnobotanical treatments for a variety of wounds and ailments, including as anthelminthics. Specifically, four *Polygonum* spp. were mentioned, with applications against ectoparasites and syphilis among other uses [5,6]. *Polygonum acuminatum* Kunth has also been reported to possess antimalarial properties [7]. Very recently, other antiparasite properties of *Polygonum* species have been reported, including anthelminthic [8,9] and antiprotozoal activities, the latter against an important parasite of fresh water fish, *Ichthyophthirius multifiliis* [10]. However, *Polygonum*-derived antiparasitics have yet to be isolated and positively identified.

This report focusses on the isolation of phytochemicals from the aerial parts of *P. salicifolium*, and their antiparasitic activities. Specifically, we tested compounds against *Trypanosoma brucei brucei*, *T. congolense,* and *Leishmania mexicana*. The *T. brucei* subspecies *T. b. gambiense* and *T. b. rhodesiense* are the etiological agents of human African trypanosomiasis [11], commonly known as sleeping sickness, whereas *T. congolense* is the most important pathogen causing the important livestock disease, nagana, in Sub-Saharan Africa [12]. *L. mexicana* is one of the parasites causing cutaneous leishmaniasis, a condition that is highly prevalent throughout the Middle East [13], whereas visceral leishmaniasis is also increasing around the Mediterranean Sea [14] and in Black Sea countries [15,16]. Drugs for these conditions are old and inadequate [17,18,19], and the current report is part of our investigations into whether new medicines can be developed from local medicinal plants [20,21,22,23] or propolis [24,25,26] as a sustainable solution for developing countries [27], and to validate ethnopharmacological practice [21].

## 2. Results

### 2.1. Isolation and Identification of Compounds

**Figure 1:**
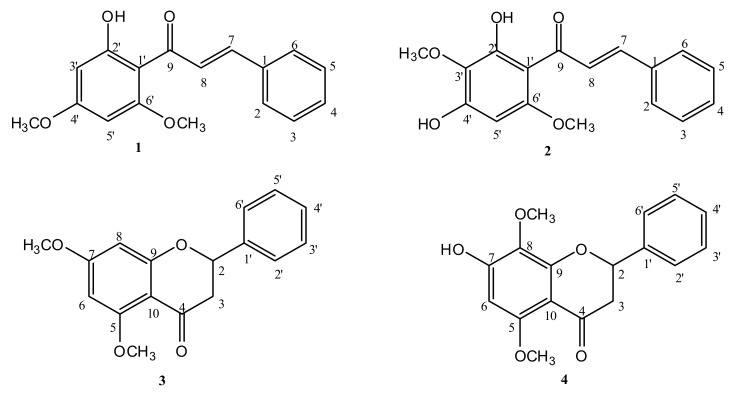
Structures of the test compounds isolated from *Polygonum salicifolium*.

The compound 2′,4′-dimethoxy-6′-hydroxychalcone (**1**) (Figure 1) was obtained as a yellow solid (50 mg) from the combined column fractions 9–11 of the hexane extract. It was purified by preparative thin layer chromatography (PTLC), using 30% EtOAc in hexane as the mobile phase. On TLC (Rf = 0.48), the compound appeared as a yellow spot, but under short UV, it appeared as a dark spot. However, the compound appeared as a brown spot after spraying with p-anisaldehyde-sulfuric acid and heating. Its mass ion [M+H]^+^ observed at *m*/*z* 285.0, suggested a molecular formula of C_17_H_16_O_4_ (Supplementary Materials Figure S1). The proton spectrum indicated the presence of seven aromatic protons, which needed to be from two phenyl rings (Supplementary Materials Figure S2). Based on integration and ^1^H-^1^H couplings in the COSY spectrum, one of the rings was tetra and the other mono substituted (Supplementary Materials Figure S3). Protons H-3, H-4, and H-5 on the mono-substituted ring appeared as a multiplets between δ_H_ 7.37 and 7.43 ppm (3H, m), while protons H-2 and H-6 appeared as a doublet at 7.62 (2H, dd, *J* = 7.6, 1.8). The signals at 3.86 (3H, s) and 3.95 (3H, s) were assigned to 4′- and 2′-OCH_3_, respectively, while the one at δ_H_ 14.30 ppm was attributed to the H-bonded or chelated 6′-OH. The meta-coupled aromatic protons at 6.14 (1H, d, *J* = 2.4) and 5.99 (1H, d, *J* = 2.4) were assigned to H-5′ and H-3′, respectively. Two trans-coupled olefinic protons were observed at δ_H_ 7.91 (1H, *d*, *J* = 15.6, α-H) and 7.83 (1H, d, *J* = 15.6, β-H). The carbon spectrum indicated the presence of 12 aromatic and two olefinic carbon signals (Supplementary Materials Figure S4). The signal at δ_C_ 192.5 was attributed to the carbonyl carbon of the chalcone, while the signals at 127.4 and 142.2 ppm were assigned to the α- and β-olefinic carbons, respectively. The rest of the signals were for the aromatic ring carbons. These assignments were further supported by the HMBC and HSQC spectra for the compound (Supplementary Materials Figures S5 and S6). The hydroxyl proton showed long range correlations (^3^*J*) to C-5′ and C-1′, and (^2^*J*) to C-6′. Hence, it must be attached at C-6′. The methoxy group protons at δ_H_ 3.95 and 3.86 showed long range correlations to the carbons at δ_C_ 162.4 (C-2′) and 166.1 (C-4′), respectively; hence, they must also be attached to these carbons. The full chemical shift assignments are given in Supplementary Table S1. It has been previously reported from Kava Plant (*Piper methysticum*) [28].

Compound **2,** identified as 2′,5′-dimethoxy-4′,6′-dihydroxychalcone (Figure 1), was also obtained as a yellow solid (40 mg) from the combined column fractions 33–37 of the hexane extract. It was also purified by PTLC using 40% EtOAc in hexane as the mobile phase. Compound **2** also appeared as a yellow spot on TLC (Rf = 0.53). Under short UV, it appeared as a dark spot, which turned into a brown spot after spraying with p-anisaldehyde-sulfuric acid, followed by heating. The positive mode HRLC-MS spectrum showed a molecular ion [M+H]^+^ at *m*/*z* 301.1100 (Calc 301.1076, C_17_H_17_O_5_), suggesting a molecular formula of C_17_H_16_O_5_ (Supplementary Materials Figure S7). The proton spectrum was similar to that of compound **1**, except for the presence of six aromatic protons (Supplementary Materials Figure S8). The difference was due to an extra substitution in the A ring, as the mono-substituted ring protons were still identical. Based on the integration and ^1^H-^1^H couplings in the COSY spectrum, this was confirmed by the disappearance of the meta-coupled protons in compound **1**, now replaced by a proton singlet in compound **2** (Supplementary Materials Figure S9). The two methoxy group signals appeared at δ_H_ 3.83 ppm (3H, s) and 3.86 ppm (3H, s), while the chelated 6′-OH was at 14.28 ppm. The aromatic singlet at δ 5.99 ppm (s) was assigned to H-3′. The trans-olefinic protons were also observed at δ_H_ 7.82 ppm (1H, d, *J* = 15.6, α-H) and 7.72 (1H, d, *J* = 15.6, β-H). The ^13^C spectrum of this compound showed 17 signals identical to compound **1**, but with one aromatic CH less and replaced by a quaternary carbon signal at 128.4 ppm (Supplementary material S10). Long range correlations in the HMBC (Supplementary Materials Figure S11), indicated the hydroxyl proton at δ_H_ 14.20 ppm showed correlations with C1′, C-6′, and C-5′, while the methoxy protons at 3.83 and 3.86 showed correlations to C-2′ (δ_C_ 158.8) and C-5′ (δ_C_ 128.4), respectively. Using these long-range correlations and the HSQC (Supplementary Materials Figure S12) spectrum, the complete chemical shift assignments (Table S2) were made. Compound **2** was previously isolated from the leaves of *P. limbatum* [29,30].

Compound **3,** identified as 5,7-dimethoxyflavanone (Figure 1), was obtained as a yellow solid (15 mg) from the combined column fractions 70–76 of the hexane extract. It was similarly purified by PTLC using 70% EtOAc in hexane as the mobile phase. On TLC (Rf = 0.37), it appeared as a dark spot when visualized under short UV and as a light blue under long UV. The spot of the compound turned yellow after spraying with p-anisaldehyde-sulfuric acid reagent followed by heating. Its mass ion [M+H]^+^ was observed at *m*/*z* 285.1, suggesting a molecular formula of C_17_H_16_O_4_ (Supplementary Materials Spectrum S13). The proton spectrum showed the presence of seven aromatic protons (Supplementary Materials Spectrum S14), which was suggested to be from a flavanone nucleus. Based on the integration and ^1^H-^1^H couplings (Supplementary Materials Figure S15) in the COSY spectrum, the proton signal at δ_H_ 5.44 ppm (1H, dd, *J* = 13.1, 2.8) showed an ABX spin system with the axial proton at δ_H_ 3.05 ppm (1H, dd, J = 16.5, 13.2) and the equatorial one at δ_H_ 2.83 ppm (1H- dd, J =16.5, 2.8). Protons H-2′, H-3′, H-4′, H-5′, and H-6′ on the mono-substituted B ring appeared as a multiplet, between δ_H_ 7.37 and 7.45 ppm (5H, m). The meta-coupled aromatic protons at δ_H_ 6.13 ppm (1H, d, *J* = 2.3) and 6.19 ppm (1H, d, *J* = 2.3) were assigned to H-6 and H-8, respectively. The signals at δ 3.85 ppm (3H, s) and 3.98 ppm (3H, s) were assigned to C-7 and C-5-OCH_3_, respectively. The ^13^C spectrum of this compound (Supplementary Materials Figure S16) showed the presence of 17 carbon atoms, corresponding to carbon atoms of the flavanone moiety and two methoxy groups. The methoxy group protons at δ_H_ 3.85 and 3.98 ppm showed long range correlations to the carbons at δc at 166.0 (C-7) and 162.3 (C-5), respectively (Supplementary Materials Figures S17 and S18); hence, they must also be attached to these carbons. These assignments were further supported by the HMBC spectrum for the compound, and the full chemical shift assignments are given in Table S3. Compound **3** was also isolated from Kava *(Piper methysticum)* roots [31].

Compound **4,** identified as 5,8-dimethoxy-7-hydroxyflavanone (Figure 1), was obtained as a white solid (10.0 mg) from the PTLC purification of fractions 90-97 of the hexane extract of *P. salicifolium*, using 10% (*v*/*v*) MeOH in EtOAc as the mobile phase. On the TLC (Rf = 0.32 in EtOAc: hexane 7:3), it appeared as a dark spot under short UV and a white spot under long UV, which turned yellow upon spraying with p-anisaldehyde-sulfuric acid reagent followed by heating. Its mass ion [M+H]^+^ at *m*/*z* 301.2 supported a molecular formula of C_17_H_16_O_5_ (Supplementary Materials Figure S19). The proton spectrum was similar to that of compound **3**, but showed six aromatic protons (Supplementary Materials Figure S20). The difference was due to an extra substitution in the A ring, whereas the mono-substituted ring protons were still identical. This was confirmed by the disappearance of the meta-coupled protons in compound **3**, now replaced by a proton singlet in compound **4**. The proton signal at δ_H_ 5.45 ppm (1H, dd, *J* = 12.9, 3.0) also showed an ABX spin system with the axial proton at δ_H_ 3.0 ppm (1H, dd, *J* = 16.6, 13.0) and the equatorial one at δ_H_ 2.83 ppm (1H, dd, *J* = 16.6, 3.1; Supplementary Materials Figure S21). The two methoxy groups appeared at δ_H_ 3.86 and 3.85 ppm. The appearance of the proton at δ_H_ 6.19 ppm as a singlet (1H, s), and the long-range coupling with a hydroxyl bearing carbon (C-7) and with the carbon bearing a methoxy group (C-5) confirmed the penta substitution of the A ring. The signals between δ_H_ 7.35 and 7.47 ppm (5H, m) were attributed to the five protons of the unsubstituted B ring. The signals at δ_H_ 3.85 ppm (3H, s) and 3.86 ppm (3H s,) were assigned to the C-5 and C-8-OCH_3_ groups, respectively. The ^13^C spectrum of the compound (Supplementary Materials Spectrum S22) showed the presence of 17 signals, corresponding to carbon atoms of the flavanone structure and two methoxy groups. Using the HMBC and HSQC spectra for the compound (Supplementary Materials Figures S23 and S24), complete chemical shift assignments (Table S4) were made. Compound **4** was previously isolated from the aerial parts of *Polygonum senegalensis* [5].

### 2.2. Anti-Kinetoplastid and Cytotoxic Activity of the Isolated Compounds

The four compounds were tested for activity against the following three kinetoplastid pathogens: *Trypanosoma brucei brucei*, *Trypanosoma congolense,* and *Leishmania mexicana*. For the two *Trypanosoma* species, the compounds were tested in parallel against strains that were resistant to the most common trypanocides. *T. b. brucei* B48 is highly resistant to the entire classes of diamidines and melaminophenyl arsenicals [28], and *T. congolense* DA-Res was rendered resistant to diminazene aceturate by means of in vitro exposure to the drug. The highest activity was observed against *T. b. brucei*, followed by *T. congolense*, but only a moderate activity was observed against *L. mexicana* (Table 1). For the drug-resistant trypanosome strains, highly significant resistance to diamidines was confirmed, but there was no cross-resistance with the chalcones and flavanones. None of the four compounds displayed measurable toxicity against the human foreskin fibroblast (HFF) cell line at the highest concentration tested (100 µg/mL; Table 1). Compound **1** was the most active against all kinetoplastid species and strains, displaying a promising EC_50_ of 0.58 µg/mL (2.04 µM) against *T. b. brucei* in our standard resazurin-based assay. Chalcone **2** was the second-most active, showing about 8-fold, 4-fold, and 5-fold less activity against *T. b. brucei*, *T. congolense,* and *L. mexicana*, respectively (Table 1). While this dataset of two chalcones is self-evidently insufficient for a structure–activity relationship (SAR) analysis, it is clear that the position of the hydroxy and methoxy groups on ring A (Figure 1) influenced the trypanocidal activity without increasing the toxicity.

## 3. Discussion

Leishmaniasis and trypanosomiasis are a heterogeneous group of neglected parasitic diseases of public health concern [29]. These diseases remain endemic in several countries worldwide [29,30,31]. The search for novel drugs remains one of the major control strategies for combating these diseases [32], as safe and effective treatment of the various forms of leishmaniasis and trypanosomiasis remains a major challenge [19,32]. The present study provides data related to the investigation into the effect of *P. salicifolium* chalcones and flavanones against *Trypanosoma* species and *Leishmania* promastigotes. This work also investigated the effect of compounds against a human cell line and cross-resistance with first line clinical trypanocides such as diamidines and melaminophenyl arsenicals. The phytochemical investigation of the hexane extract of *P. salicifolium* led to the isolation of two chalcones—compound (**1**), and compound (**2**)—and two flavanones—compound (**3**) and compound (**4**). This is an initial report of their isolation from *Polygonum salicifolium*.

The anti-kinetoplastid activities of the compounds isolated from *P. salicifolium* indicate that flavanones **3** and **4** displayed a moderate activity against *Trypanosoma* species, and their activity against *Leishmania* promastigotes was poor. However, chalcone **1** displayed a promising activity against *T. b. brucei* at ~2 µM, as well as a moderate activity against *T. congolense* and a reasonable activity against *L. mexicana*. No cross-resistance with the current trypanocides was observed, which is very important, as resistance to the old anti-kinetoplastid drugs is a key driver of the need for new treatments [11,12,18]. The consistently lower anti-protozoal activity of chalcone **2** suggests that a systematic investigation of the structure–activity relationship of chalcones, including substitutions on the chalcone rings, could yield compounds with substantially improved efficacy against parasites. Importantly, neither of the chalcones showed toxicity against the human cell line HFF at 100 µg/mL, and the in vitro selectivity index of 1 was >172. An important advantage of the chalcones over the flavanones is that their synthesis, and thus SAR, should be more accessible than that of most natural compounds because of the lack of chiral centers [33].

## 4. Materials and Methods

### 4.1. General Experimental Procedures

The ^1^H and ^13^C NMR spectra were run on a Bruker AVANCE III 500 MHz spectrophotometer, operating at 500 MHz (^1^H) and 125 MHz (^13^C), respectively, using CDCl_3_ as the solvent and TMS as the internal standard. The mass spectra were recorded using a Thermo LTQ Orbitrap, while the exact masses were determined using a Thermo Exactive Orbitrap mass spectrometer. Column chromatographic separations were performed in glass columns using silica gel MN-60 (Macherey-Nagel GmbH, KG, Düren, Germany). TLC and PTLC were carried out using pre-coated silica gel 60 Aluminium sheets (Merck Chemicals, Bedfont Lakes Business Park Feltham, U.K.). The spots on the TLC were visualized using an anisaldehyde-H_2_SO_4_ reagent. Solvents, hexane, ethyl acetate, and methanol were purchased from Sigma-Aldrich, U.K.

### 4.2. Collection of Plant Material

The plant *P. salicifolium* Brouss ex Wild was collected from the banks of the River Tigris in Southern Iraq in April 2015, and was identified and deposited at the College of Science, University of Diyala by Assist. Prof. Dr Khazal Dh. Wadi Al-Jibouri.

### 4.3. Preparation of Extracts

The aerial parts of the plant were dried and finely powdered with an IKA grinder (IKA Werke GmbH and Co. KG, Staufen im Breisgau, Germany). The ground material (50 g) was extracted (500 mL, 72 h each) with hexane, ethyl acetate (EtOAc), and then methanol (MeOH) using a Soxhlet apparatus. The extracts were then filtered and dried at 40 °C using a rotary evaporator (Büchi, Flawil, Switzerland).

### 4.4. Isolation and Identification of Compounds

The hexane extract (1 g; 2% yield) was subjected to silica gel column chromatography eluting gradient wise with hexane, followed by increasing amounts (10–90% *v*/*v*) of EtOAc in hexane and then EtOAc (100%). A total of 150 fractions (5 mL each) were collected and, based on TLC results, similar fractions were combined. Further purification of the compounds was carried out using preparative thin layer chromatography (PTLC) with 30–70% (*v*/*v*) EtOAc in hexane and 10% (*v*/*v*) MeOH in EtOAc. The characterization of the compounds was carried out using NMR (1D and 2D) on a Bruker AVIII HD 500 spectrophotometer using 5–20 mg samples dissolved in chloroform-*d*. The ethyl acetate and methanol extracts did not yield any significant results in the preliminary assays and spectroscopic analysis, and were thus not purified any further.

### 4.5. Parasites and Cultures

*T. b. brucei* strain Lister 427 (427-WT) was used as the standard drug-sensitive (wild-type; WT) laboratory strain [34]. This strain was previously adapted for multi-drug resistance by deletion of the TbAT1/P2 drug transporter [35], subsequently followed by in vitro exposure to increasing concentrations of pentamidine [28]. For *T. congolense*, the culture-adapted Savannah strain IL3000 was used [22], as well as a clonal line, 6C3, adapted from IL3000 in vitro to diminazene aceturate (Sigma), leading to a ~10-fold resistance. All *Trypanosoma* strains were cultured and used as bloodstream forms; *T. b. brucei* in a full HMI-9 medium supplemented with 10% Foetal Bovine Serum (FBS) [27] and *T. congolense* in TC-BSF1 medium with 20% goat serum at 34 °C, as described by Coustou et al. [36]. The promastigotes of *L. mexicana* strain MNY/BZ/62/M379 were cultured at 25 °C in a minimal essential medium, HO-MEMO-MEME, supplemented with 10% FBS and 1% of a penicillin/streptomycin solution (Gibco), as described previously [37,38].

### 4.6. Anti-Protozoal Drug Testing

The anti-trypanosomal activity of the compounds was tested using the Alamar Blue (resazurin) assay in plastic 96-well plates, as described [39]. The assay was based on the reduction of blue, non-fluorescent resazurin sodium salt (Sigma) by live, but not by dead cells, to the red fluorescent metabolite resorufin [40]. Briefly, dilutions of the test compounds and control drugs were distributed in the first wells of the respective plate rows, and doubling dilution was carried out over two rows in the appropriate medium for *T. b. brucei* or *T. congolense*, leaving the last rows as the drug-free negative control (i.e., 23 doubling dilutions). Then, 10^5^ trypanosomes were added to each well, followed by incubation of the plates at 37 °C/5% CO_2_ (*T. b. brucei*) or 34.5 °C/5% CO_2_ (*T. congolense*) for 48 h before the addition of the resazurin dye (20 µL of 125 mg/L), and a further incubation under the same conditions for 24 h. The *T. congolense* IL3000 WT and the diminazene resistant *T. congolense* IL3000 DA-Res (previously adapted to diminazene; clone 6C3) were utilized. Fluorescence was measured using a FLUOstar Optima plate reader at excitation and emission wavelengths of 544 nm and 590 nm, respectively, and the EC_50_ of the compounds was then calculated using GraphPad Prism 5, using an equation for a sigmoid curve with a variable slope. The assay for *L. mexicana* promastigotes was performed as for *T. b. brucei* [41], except that incubation times of 72 h and 48 h were used before and after the addition of resazurin, respectively, because of the slower metabolism of the dye by *Leishmania* promastigotes [40].

### 4.7. Drug Toxicity Assay

Toxicity of drugs to mammalian cells was carried out in the human cell line HFF, using a previously described method [41], with slight modifications. Briefly, HFF cells were grown in a medium consisting of 500 mL Dulbecco’s Modified Eagle’s Medium (DMEM; Sigma), 50 mL new-born calf serum (NBCS; Gibco, Cleveland, TN, USA), 5 mL penicillin/streptomycin (Gibco), and 5 mL L-Glutamax (200 mM, Gibco), at 37 °C/5% CO_2_ up to ~80% confluence in vented flasks. For the assay, 100 µL of the cell suspension (3 × 10^5^ cells/mL) was added to each well of a 96-well plate. The plate was incubated at 37 °C/5% CO_2_ for 24 h to allow for cell adhesion, after which 100 µL of a serial drug dilution was added (prepared in a separate sterile plate). Phenylarsine oxide (PAO; Sigma) was used as the positive control. The cells were then incubated for a further 30 h before the addition of 10 µL of 125 mg/L resazurin solution, and underwent a final incubation for 24 h. Fluorescence measurements and data analysis were performed, as described above. The selectivity index was calculated as EC_50_ (HFF)/EC_50_(427-WT).

## 5. Conclusions

The present data showed that chalcones exhibited interesting activity against *T. b. brucei*; *T. congolense;* and, to a lesser extent, *L. mexicana,* and provide further evidence for the potential uses of natural plant extracts for combating these global parasitic diseases. Future work should concentrate on exploring the SAR of anti-protozoal chalcones and identifying their cellular targets. In addition, future research should test for the potential activity of *P. salicifolium* extracts against different protozoan species of medical and veterinary importance.

## Figures and Tables

**Table 1:** Anti-kinetoplastid effects of the tested chalcones and flavanones.

		*T. b. brucei* EC_50_ (µM)		*T. congolense* EC_50_ (µM)		*L. mexicana* EC_50_ (µM)	HFF	
Compound	MW	427-WT	B48	IL3000-WT	DA-Res	WT	EC_50_ (µM)	SI (*Tbb*)
1	284.3	2.04 ± 0.07	1.80 ± 011	8.8 ± 0.39	8.8 ± 0.28	18.2 ± 1.0	>350	>172
2	300.3	14.6 ± 0.80	13.9 ± 0.60	34.0 ± 1.2	28.6 ± 1.4 *	83.6 ± 0.7	>330	>22.8
3	284.3	30.1 ± 0.95	30.3 ± 1.1	137 ± 10	106 ± 15	271 ± 31	>350	>11.7
4	300.3	55.3 ± 1.6	51.9 ± 1.6	63.3 ± 7.9	48.0 ± 6.2	338 ± 9	>330	>6.0
Diminazene		0.15 ± 0.01	2.4 ± 0.36 **	0.15 ± 0.01	1.43 ± 0.03 ***	ND	ND	--
Pentamidine		0.0034 ± 0.0004	0.72 ± 0.03 ***	0.72 ± 0.07	ND	0.56 ± 0.07	ND	--
PAO		ND	ND	ND	ND	ND	1.31 ± 0.08	--

All of the data listed are the average and standard error of the mean (SEM) of at least three independent determinations. PAO—phenylarsine oxide; ND—not done. Statistical significance was determined using an unpaired Student’s *t*-test; * *p* < 0.05; ** *p* < 0.01; ***, *p* < 0.001. Selectivity index (SI) = EC_50_(HFF)/EC_50_(Tbb).

## Supplementary Materials

The following are available online at https://www.mdpi.com/2076-0817/10/2/175/s1: Figures Spectrum S1–S24 and Tables S1–S4.
