# Supplementary material for: Antitrypanosomal and Antileishmanial Activity of Chalcones and Flavanones from Polygonum salicifolium
Source: Pathogens. 2021 Feb 5;10(2):175. doi: 10.3390/pathogens10020175 (PMC7915666; doi:10.3390/pathogens10020175)
Supplement: Supplementary file 1 [file pathogens-10-00175-s001.pdf]

SUPPLEMENTARY MATERIAL

Title: Antitrypanosomal and Antileishmanial Activity of Chalcones and Flavanones from *Polygonum salicifolium*

Ahmed M. Zheoat [1,7], Samya Alenezi [1], Ehab Kotb Elmahallawy [2,3], Marzuq A. Ungogo [2,4], Ali H. Alghamdi [2,5], David G. Watson [1], John O. Igoli [1,6], Alexander I. Gray [1], Harry P. de Koning [2*] and Valerie A. Ferro [1]

[1] Strathclyde Institute of Pharmacy and Biomedical Sciences, University of Strathclyde, Glasgow, United Kingdom. 1; ahmed.zheoat@strath.ac.uk (A.M.Z); s-alenezi@strath.ac.uk (S.A); igolij@gmail.com (J.O.I); .G.Watson@strath.ac.uk (D.G.W); a.i.gray345@gmail.com; (A.I.G) and v.a.ferro@strath.ac.uk (V.A.F)

2 Institute of Infection, Immunity and Inflammation, College of Medical, Veterinary and Life Sciences University of Glasgow, Glasgow, United Kingdom. 2; eehaa@unileon.es (E.K.E); 2226184U@student.gla.ac.uk (M.A.U) ; a.alghamdi.2@research.gla.ac.uk (A.H.A) and Harry.De-Koning@glasgow.ac.uk (H.P.d-k)

[3] Department of Zoonoses, Faculty of Veterinary Medicine, Sohag University, Sohag, Egypt. 3; eehaa@unileon.es (E.K.E)

[4] Department of Veterinary Pharmacology and Toxicology, Ahmadu Bello University, Zaria, Nigeria. 4; 2226184U@student.gla.ac.uk

[5] Biology Department, Faculty of Science, Albaha University, Albaha, Saudi Arabia. 5; a.alghamdi.2@research.gla.ac.uk

[6] Phytochemistry Research Group, Department of Chemistry, University of Agriculture, Makurdi, Nigeria. 6; igolij@gmail.com

[7] Al-Manara College for Medical Sciences, Misan, Iraq.

*, Correspondence:
Harry P. de Koning
Email:Harry.De-Koning@glasgow.ac.uk

Compound **1**

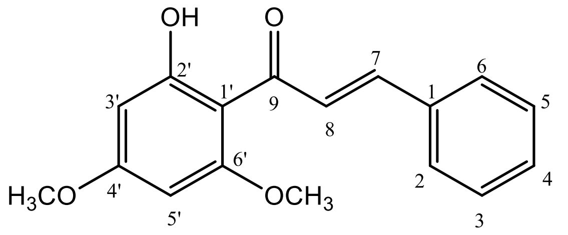

4′,6′-dimethoxy-2′-hydroxychalcone

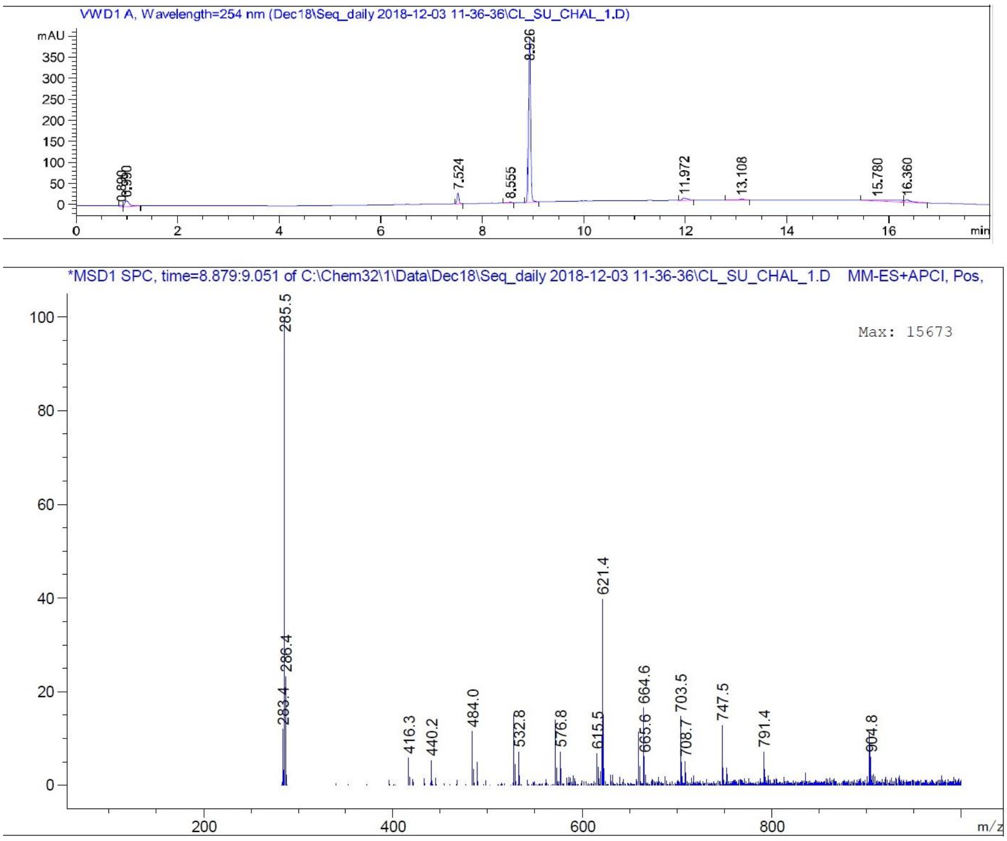

Supplementary material S1: LC-MS spectrum showing $[M+H]^+$ ion for compound **1**.

A

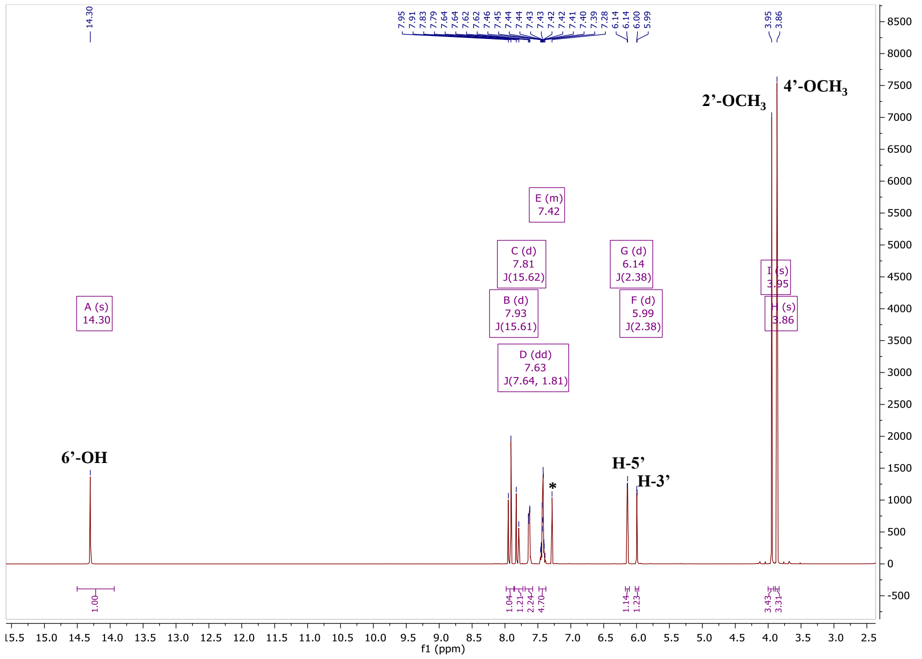

B

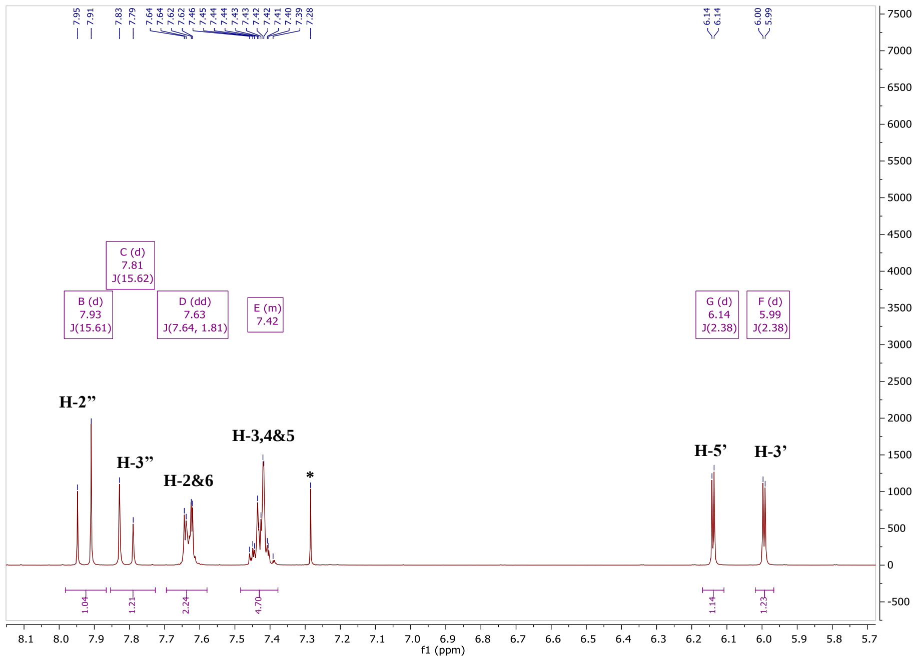

Supplementary material S2: (A) Full and (B) expansion of ^{1}H NMR spectrum (500 MHz) of compound **1** in $CDCl_3$. *Denotes residual solvent peak.

A

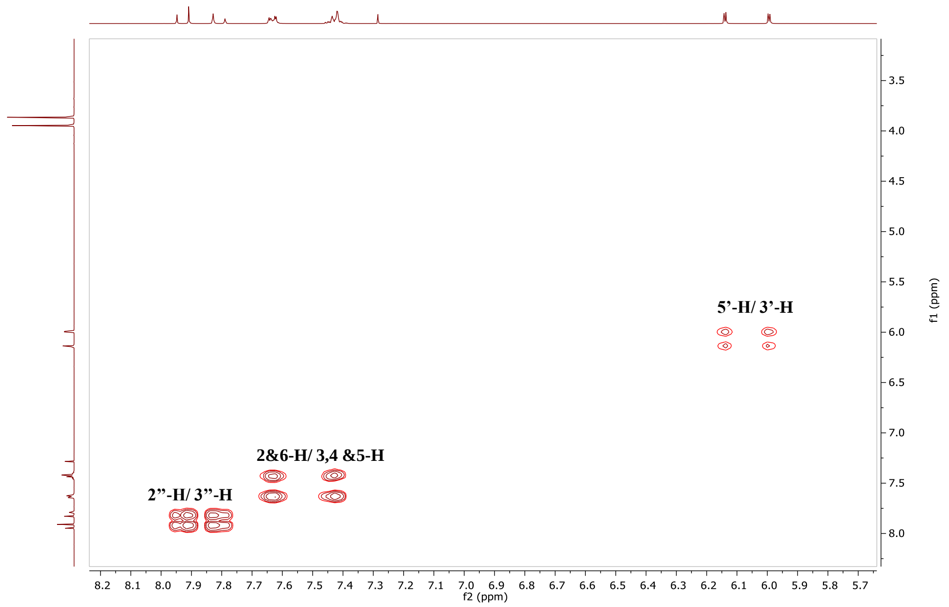

B

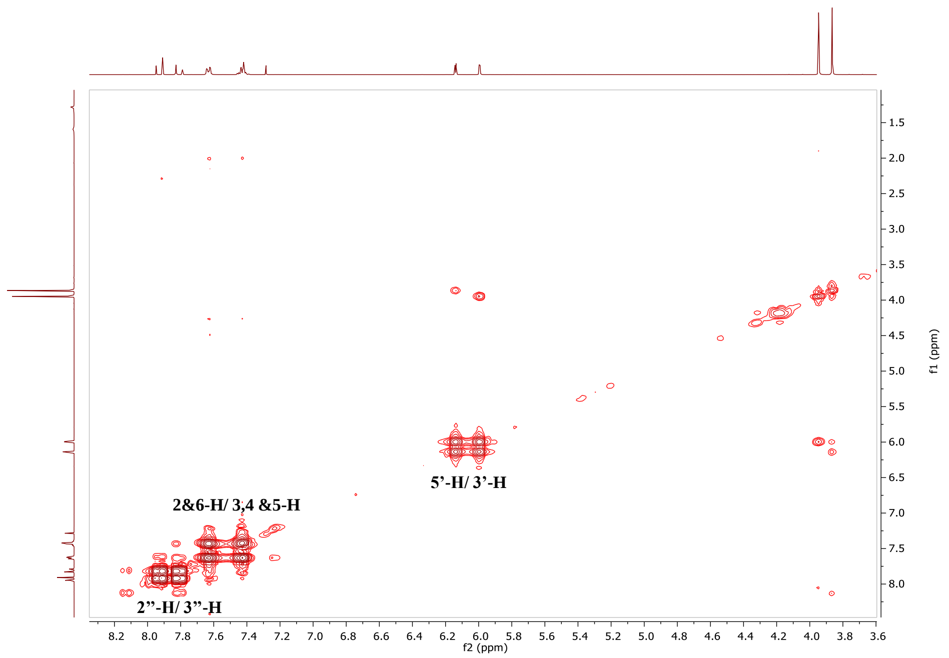

Supplementary material S3: (A) Full and (B) expansion of COSY spectrum of compound **1** in $CDCl_3$.

A

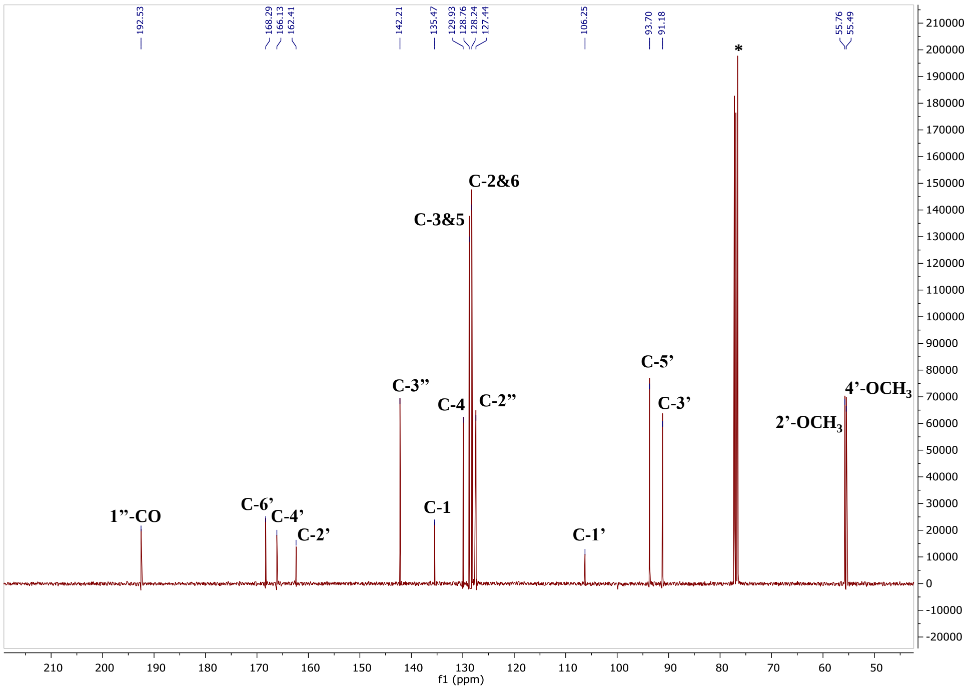

B

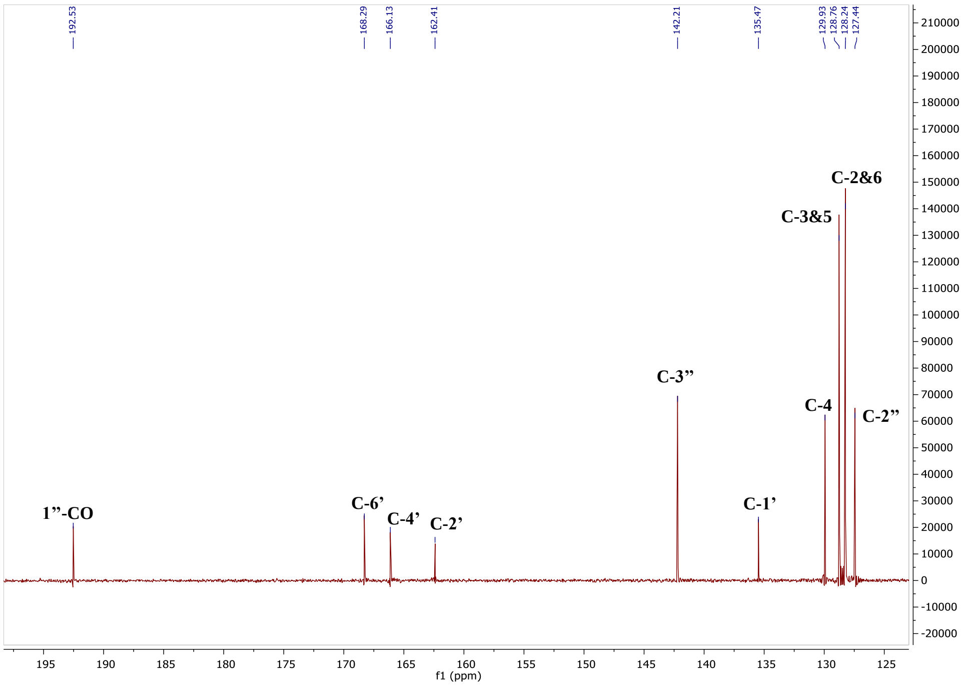

Supplementary material S4: (A) Full and (B) expansion of ^{13}C NMR spectrum of compound **1** in $CDCl_3$. *Denotes residual solvent peak.

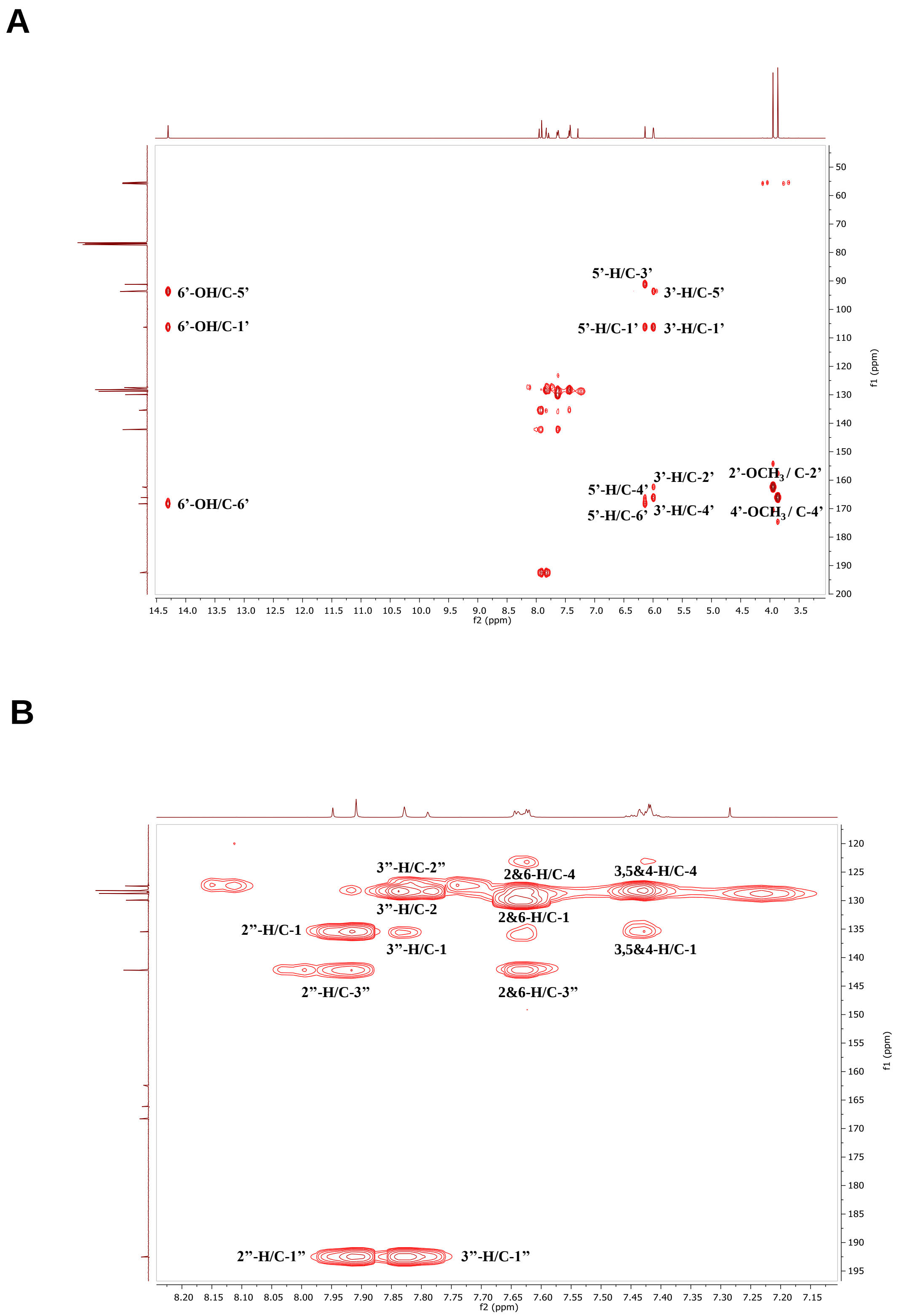

Supplementary material S5: (A) Full and (B) expansion of HMBC spectrum of compound **1** in $CDCl_3$.

A

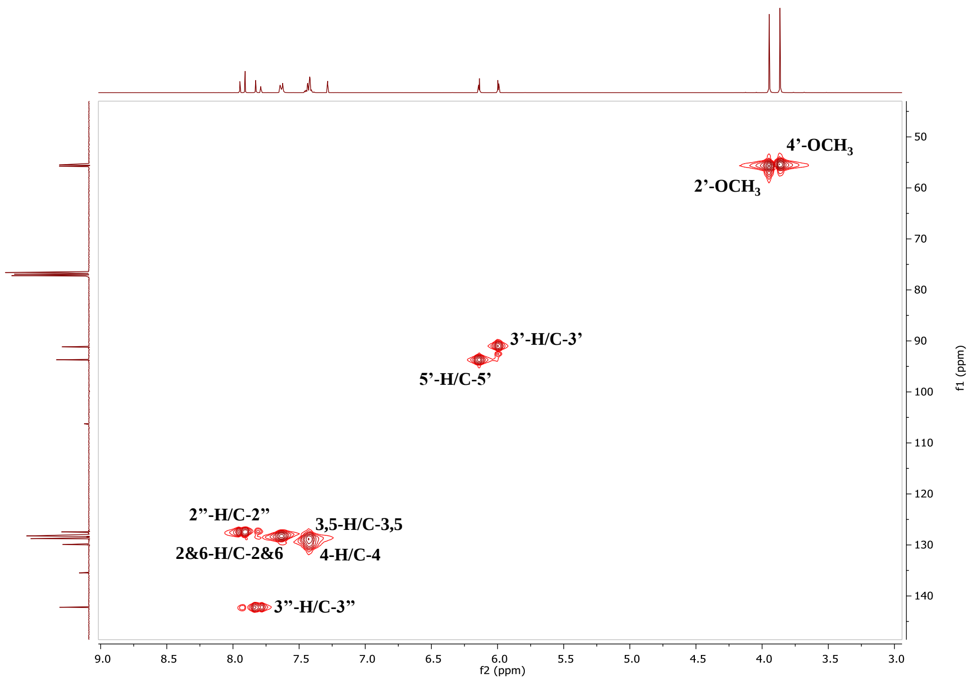

B

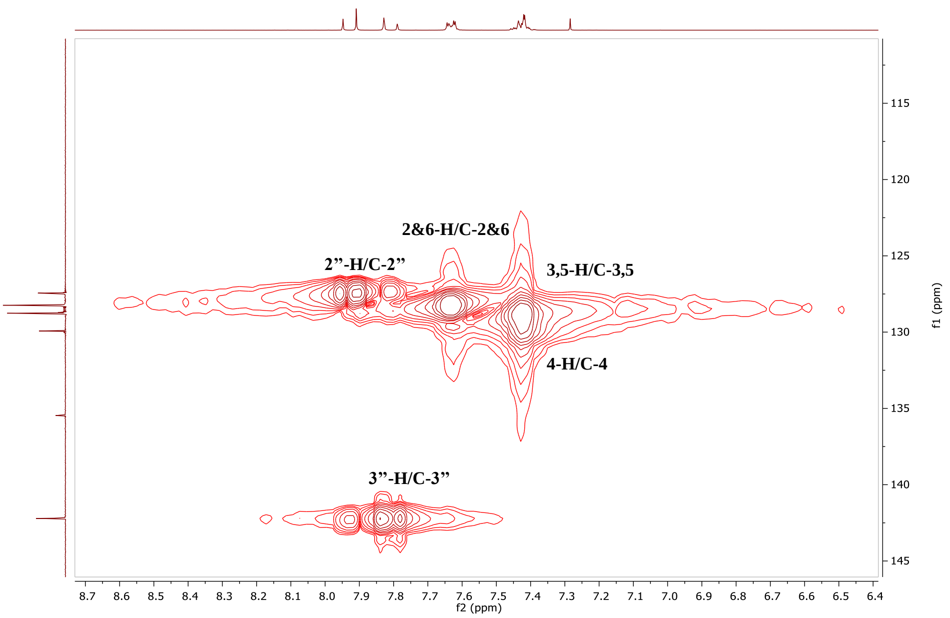

Supplementary material S6: (A) Full and (B) expansion of HSQC spectrum of compound **1** in $CDCl_3$.

Supplementary Table S1: NMR data for compound 1 in $CDCl_3$

Position	δ_H (mult, *J* (Hz))	δ_C	HMBC correlations
1	-	135.4	-
2, 6	7.62 (2H, *dd*, 7.6, 1.8)	128.2	C-1, C-4
3, 4, 5	7.42 (3H, m)	128.7 &129.9	C-1, C-4
7	7.83 (1H, d, 15.6)	142.2	C-1, C-2, C-8, C-9
8	7.91 (1H, d, 15.6)	127.4	C-1, C-7, C-9
9	-	192.5	-
1′	-	106.2	-
2′	-	168.2	-
3′	6.14 (1H, d, 2.6)	93.7	C-1′, C-2′, C-4′, C-5′
4′	-	166.1	-
5′	5.99 (1H, d, 2.6)	91.1	C-1′, C-3′, C-4′, C-6′
6′	-	162.4	-
2′-OH	14.30 (s)	-	C-1′, C-2′, C-3′
O-CH_3	3.86 (3H, s)	55.4	C-4′
O-CH_3	3.95 (3H , s)	55.7	C-6′

Compound **2**

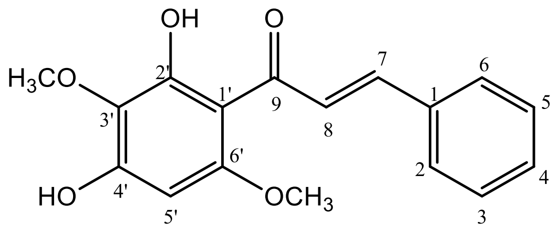

3′,6′-dimethoxy-2′,4′-dihydroxychalcone.

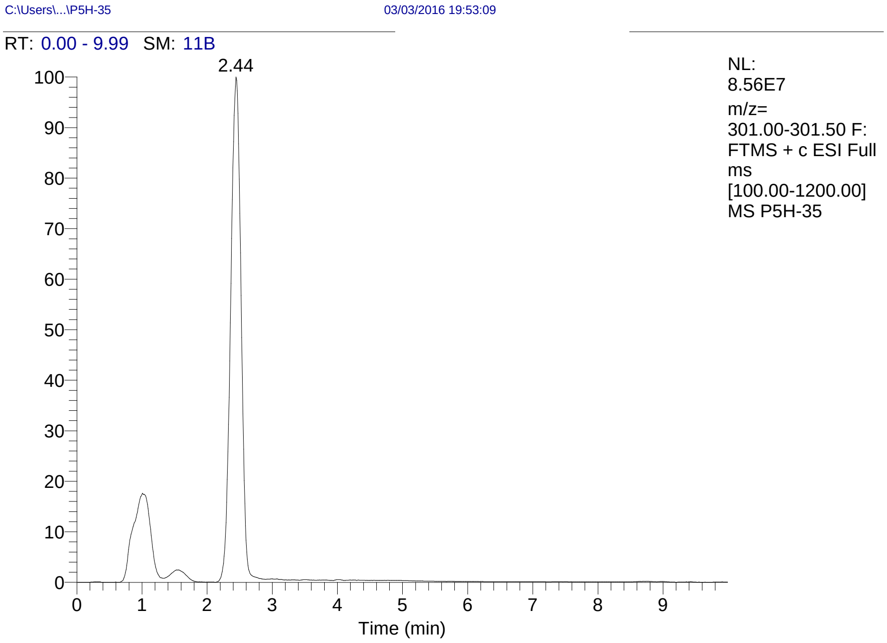

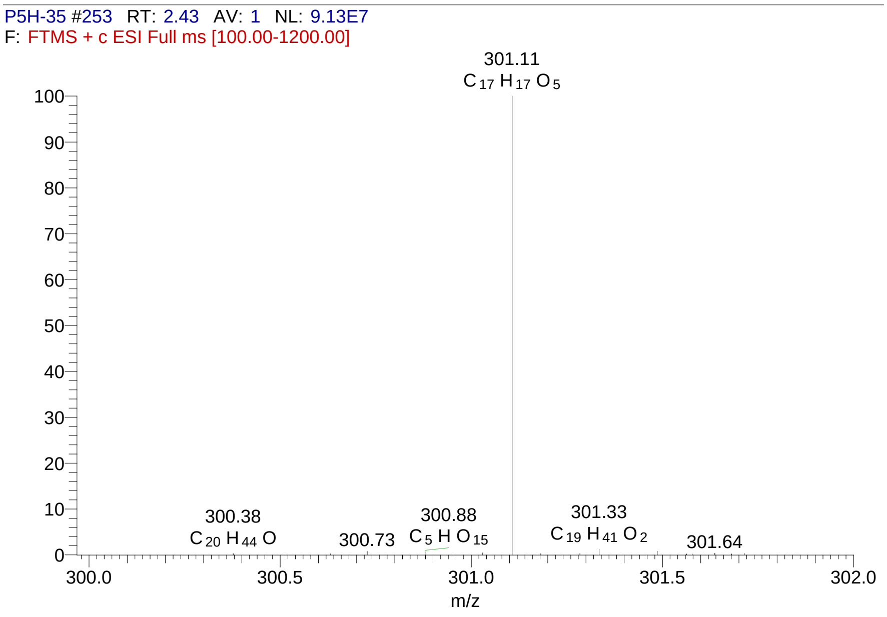

Supplementary material S7: HR-LC-MS spectrum showing $[M+H]^+$ ion for compound **2**

A

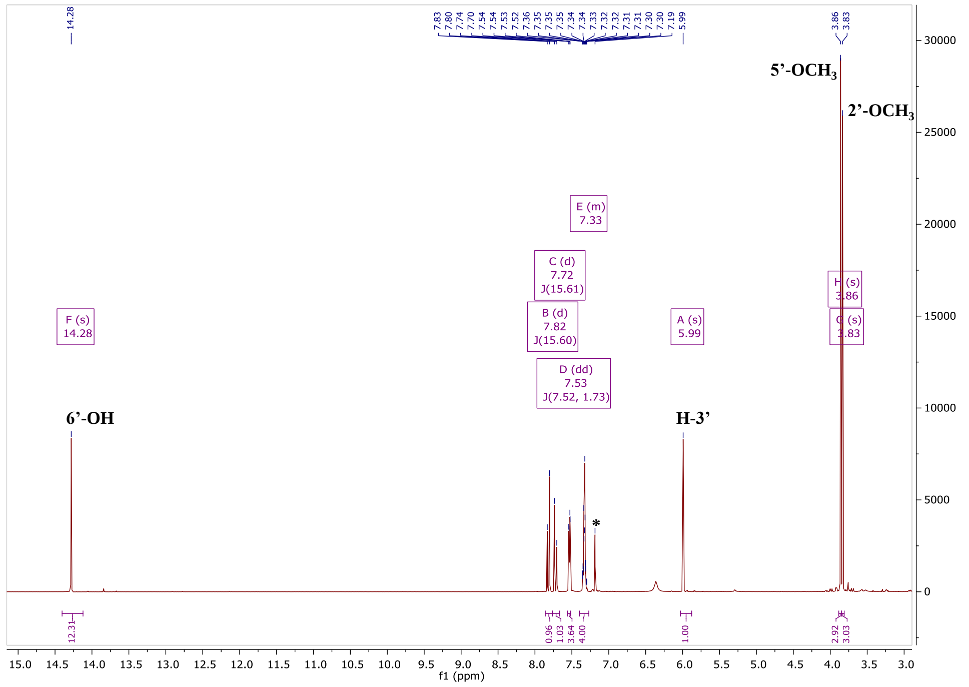

B

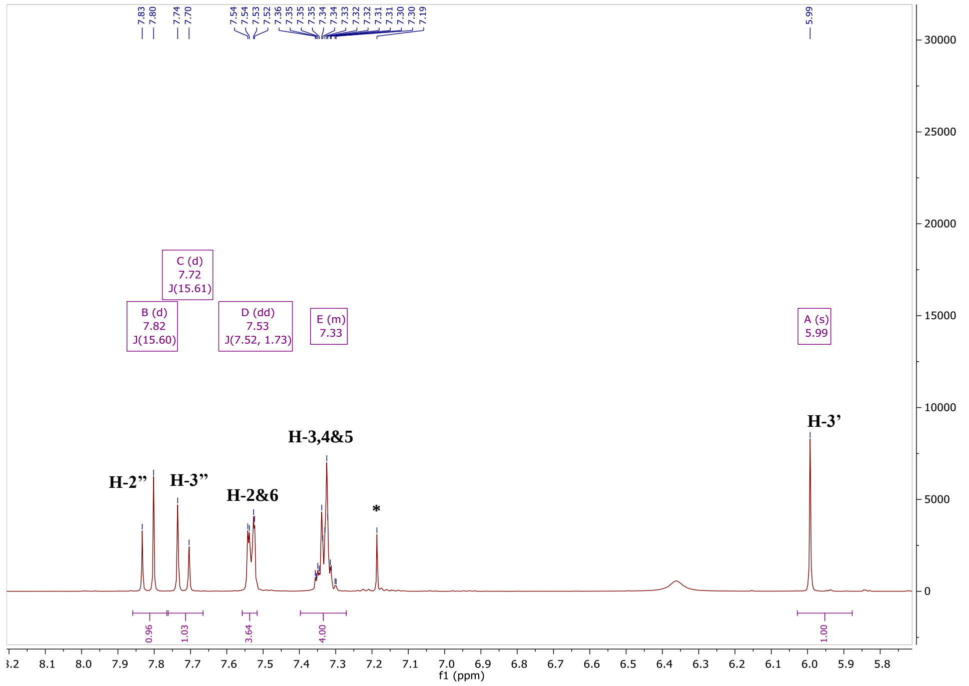

Supplementary material 8: (A) Full ^{1}H NMR spectrum (500 MHz) of compound **2** in $CDCl_3$ and (B) expansion of the aromatic region. *Denotes residual solvent peak.

A

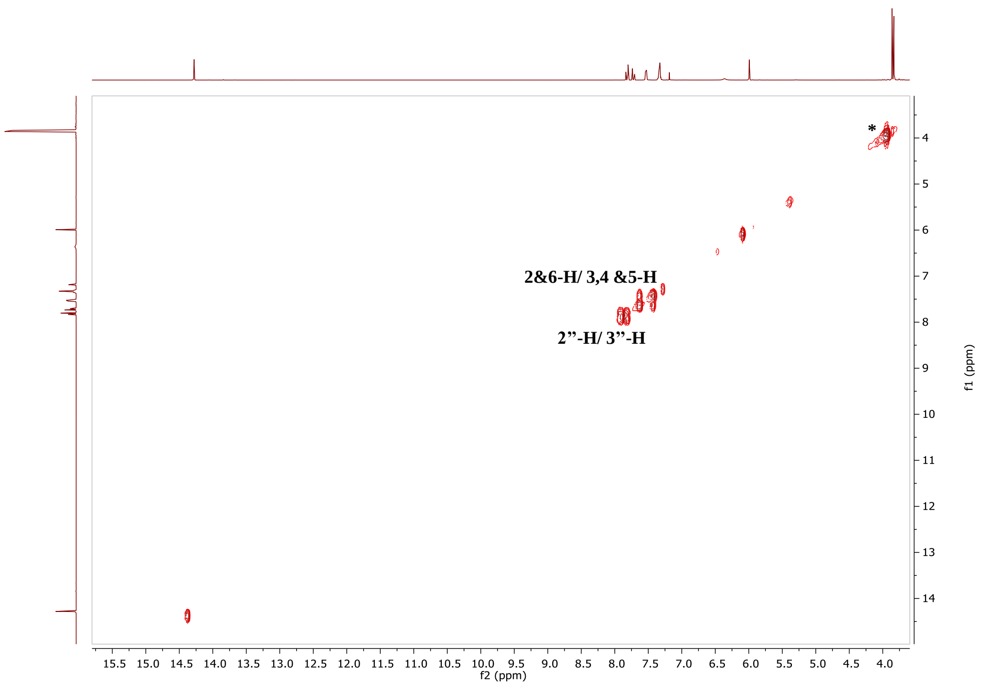

B

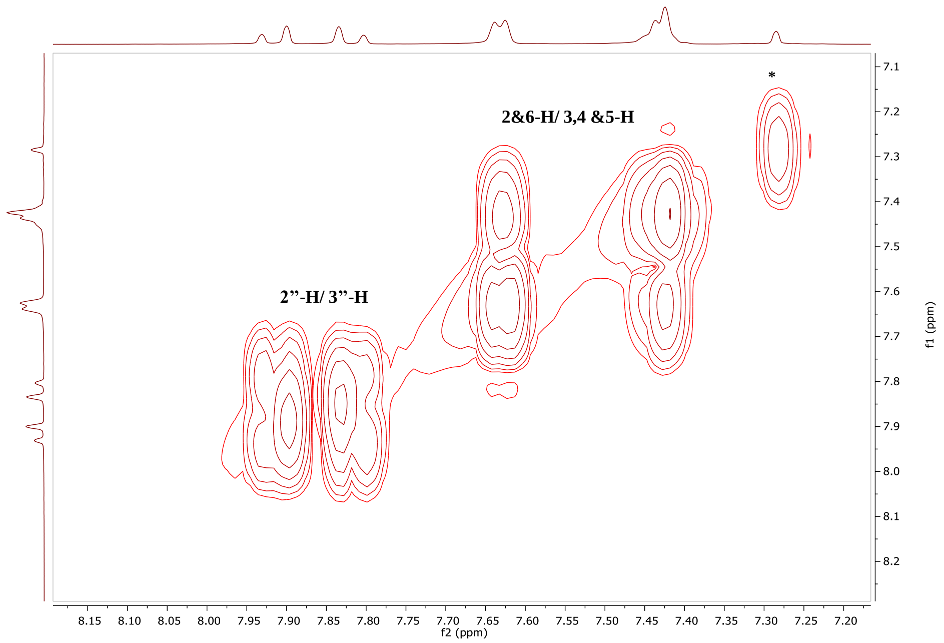

Supplementary material S9: (A) Full and (B) expansion of COSY spectrum of compound **2** in $CDCl_3$.

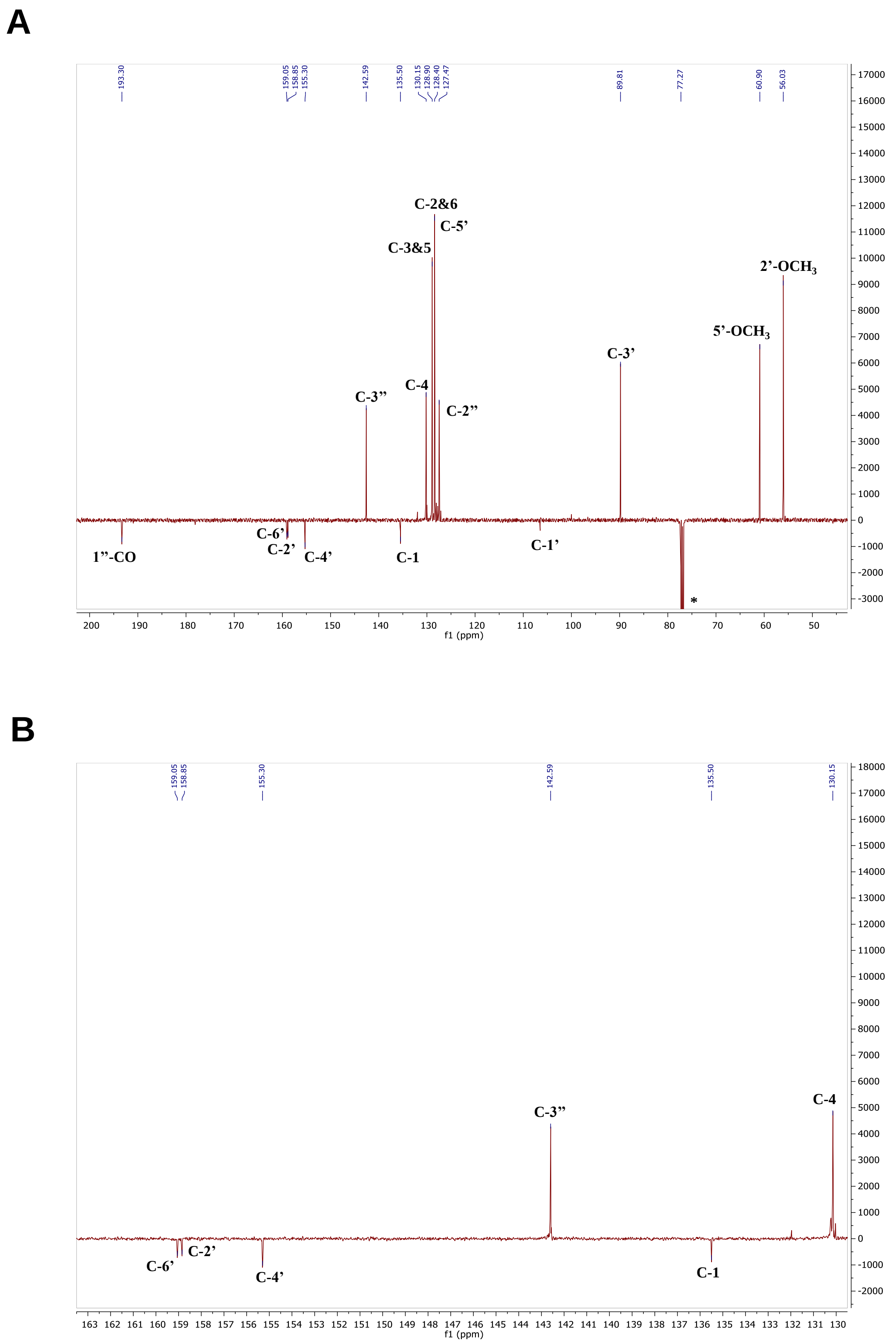

Supplementary Spectrum S10: (A) Full and (B) expansion of DEPTq 135 ^{13}C NMR spectrum of compound **2** in $CDCl_3$. *Denotes residual solvent peak.

A

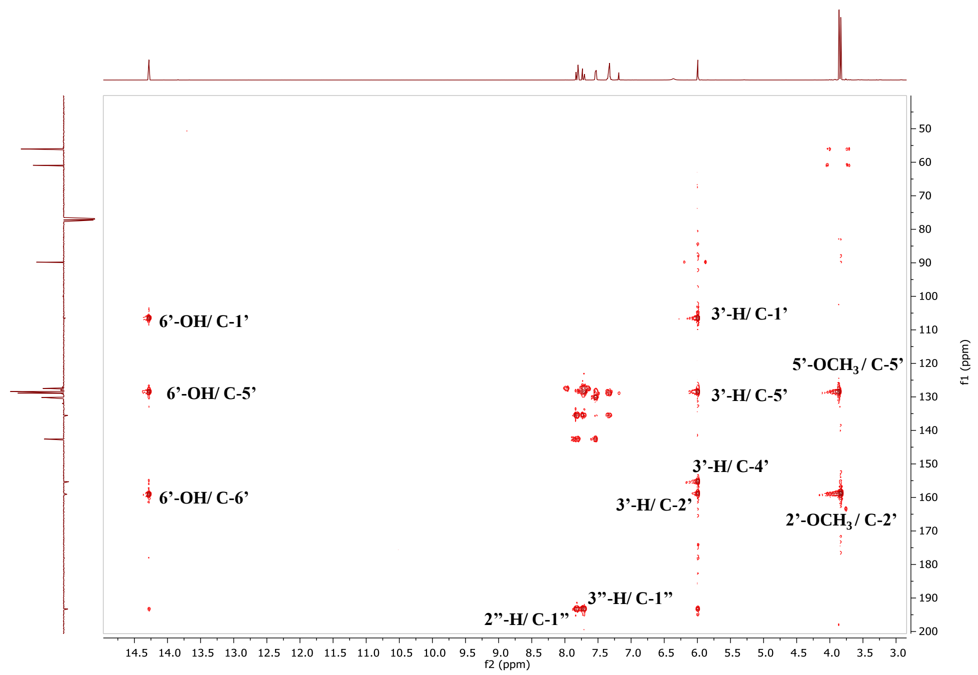

B

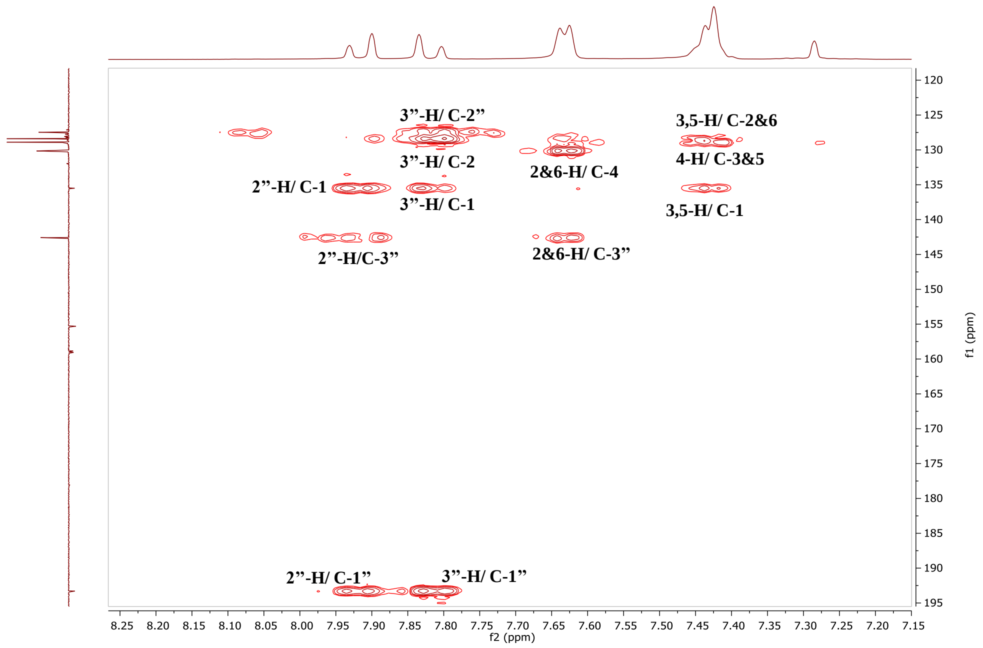

Supplementary material S11: (A) Full and (B) expansion of HMBC spectrum of compound **2** in $CDCl_3$.

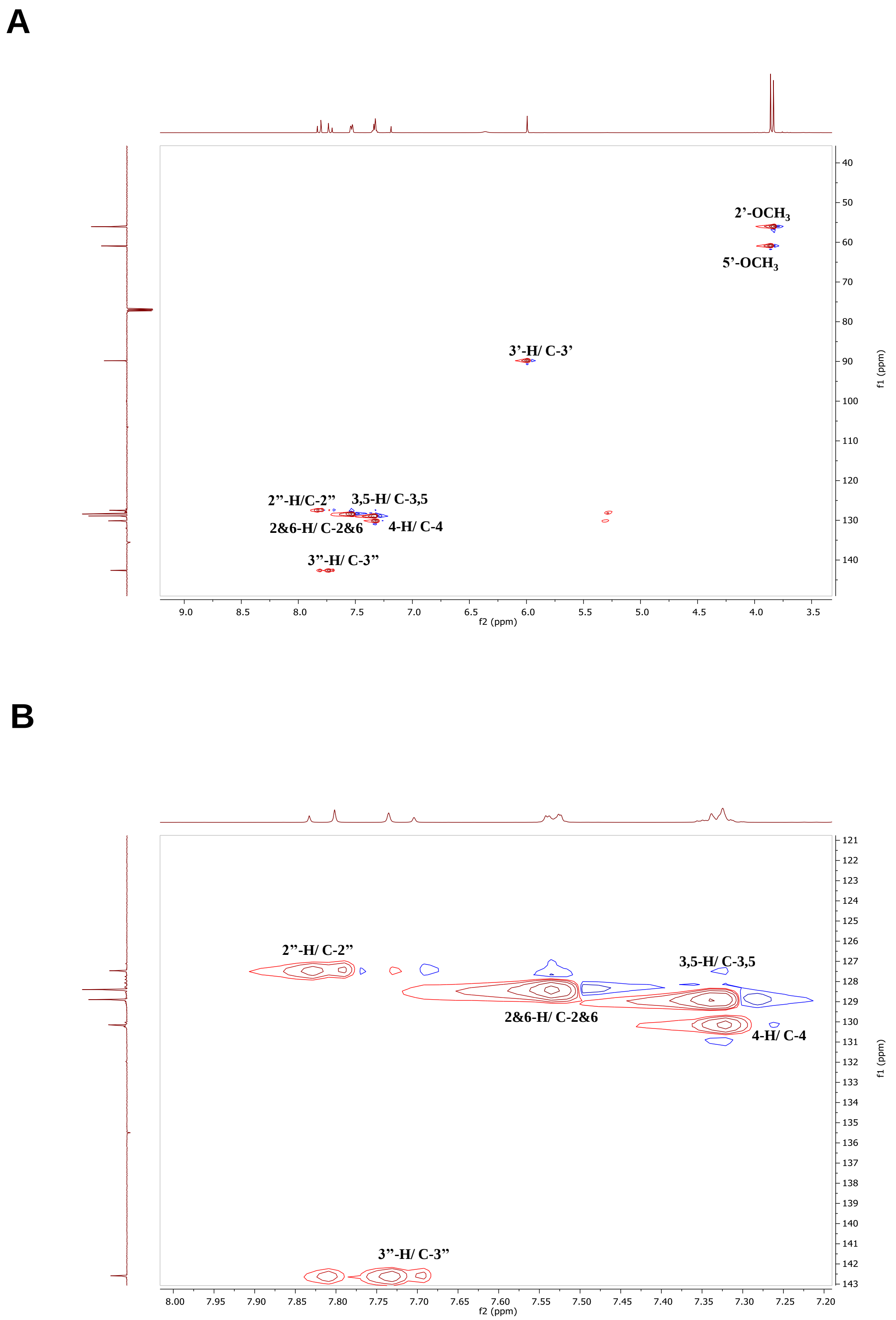

Supplementary material S12: (A) Full and (B) expansion of HSQC spectrum of compound **2** in $CDCl_3$.

Supplementary Table S2: NMR data for compound 2 in $CDCl_3$

**Position**	**δ_H (mult, *J* (Hz))**	**δ_C**	**HMBC**
1	-	135.5	-
2, 6	7.53 (2H, dd, 7.5, 1.7)	128.4	C-1, C-7
3, 4, 5	7.33 (3H, m)	128.9, 130.1	C-1, C4
7	7.72 (1H, d, 15.6)	142.5	C-1, C-9, C-8
8	7.82 (1H, d, 15.6)	127.4	C-1, C-9, C-7
9	-	193.3	-
1′	-	106.5	-
2′	-	159.0	C-1′, C-2′, C-3′
3′	-	128.4	-
4′	-	155.3	-
5′	5.99 (1H, s)	89.8	C-1′, C-3′, C-4′, C-6′
6′	-	158.8	-
2′-OH	14.2 (s)	-	-
O-CH_3	3.83 (3H, s)	60.1	C-4′
O-CH_3	3.86 (3H , s)	56.0	C-6′

Compound **3**

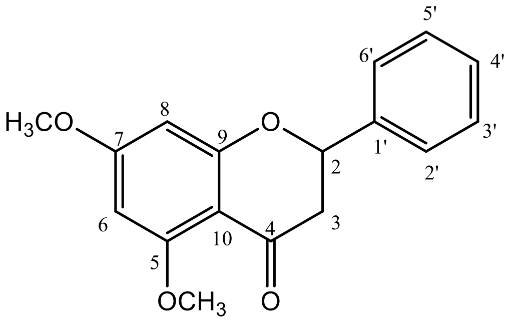

5,7-dimethoxyflavanone

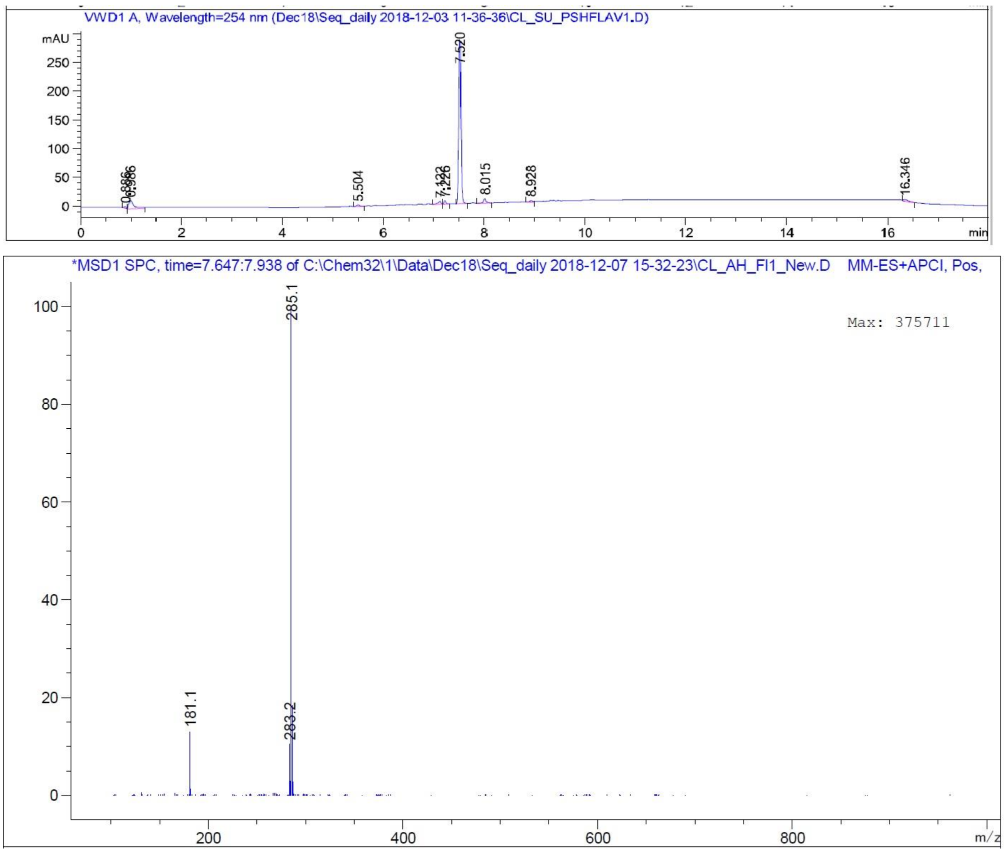

Supplementary material S13: LC-MS spectrum showing $[M+H]^+$ ion for compound **3**.

A

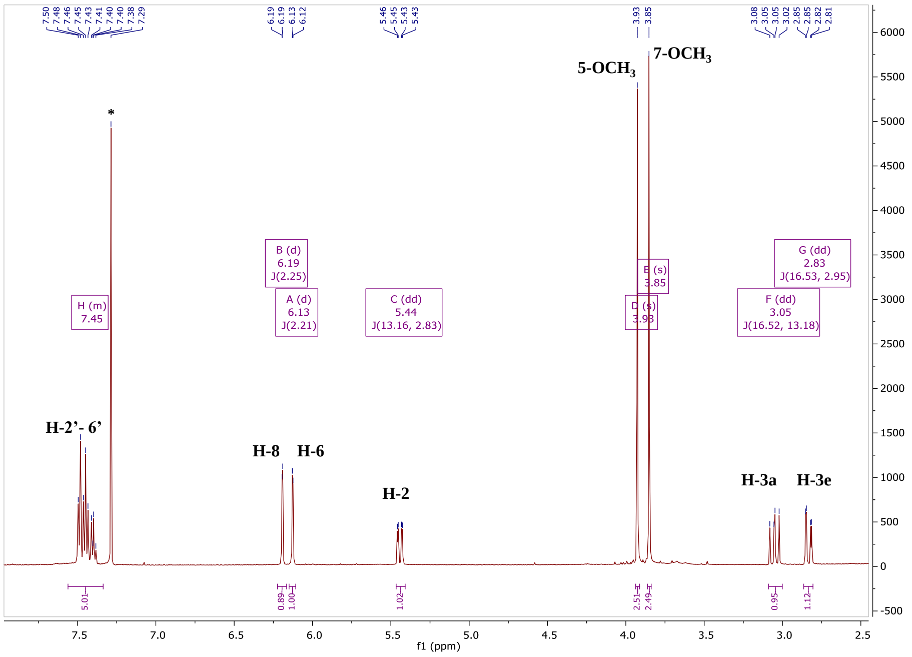

B

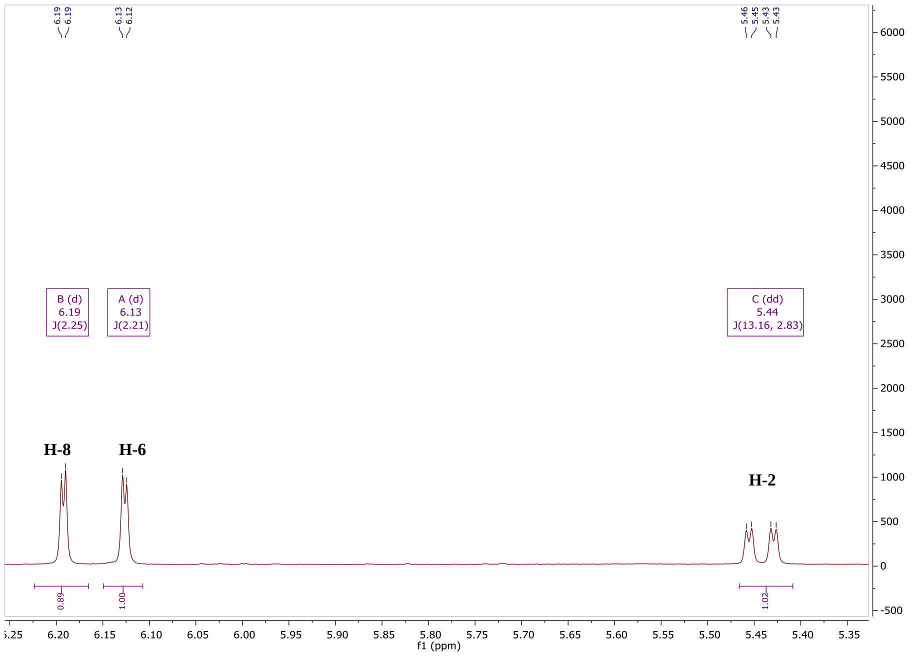

Supplementary material S14: (A) Full and (B) expansion of ^{1}H NMR spectrum (500 MHz) in $CDCl_3$ of compound **3**. *Denotes residual solvent peak

A

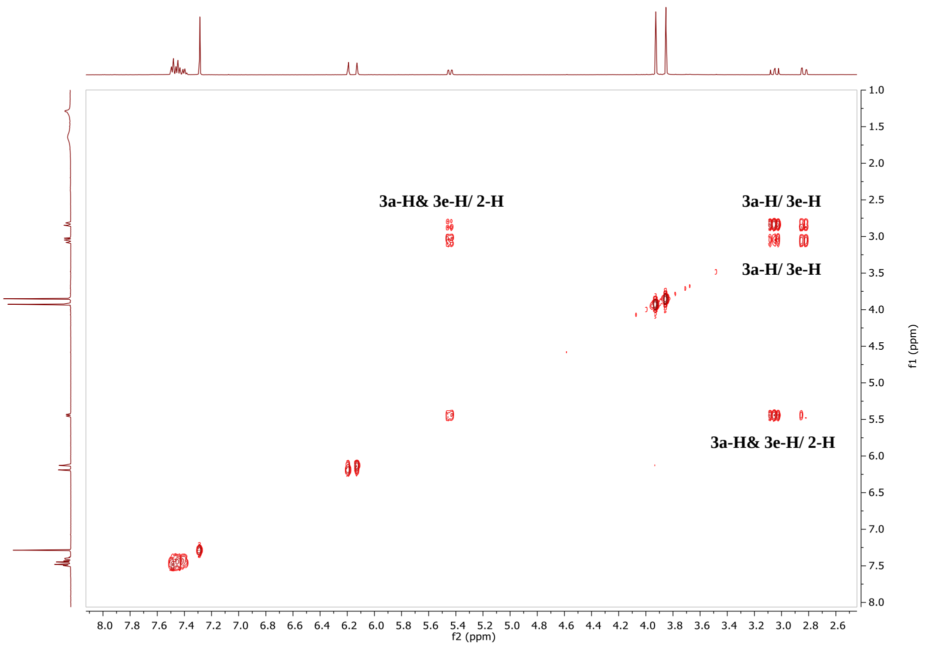

B

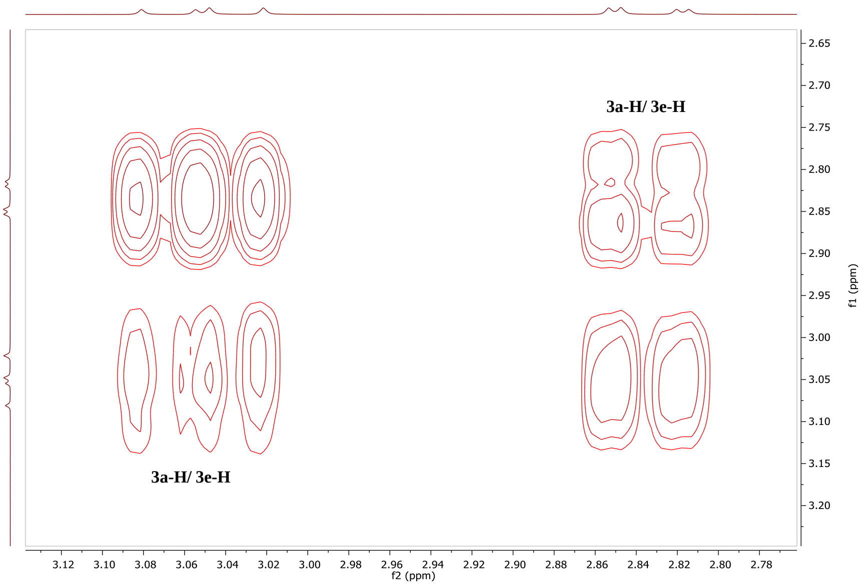

Supplementary Spectrum S15: (A) Full and (B) expansion of COSY spectrum of compound **3** in $CDCl_3$.

A

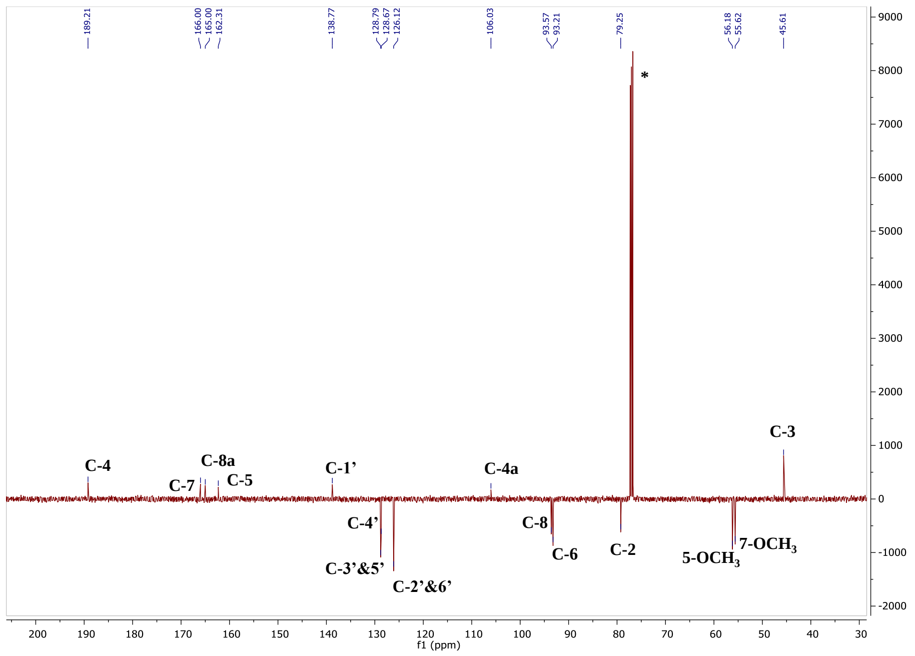

B

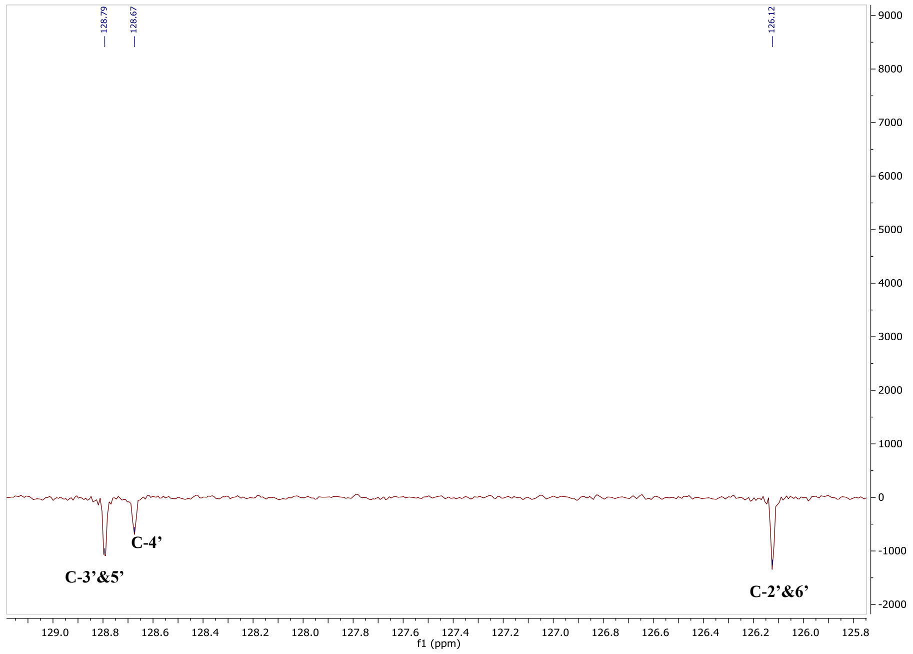

Supplementary material S16: (A) Full and (B) expansion of DEPTq 135 ^{13}C NMR spectrum of compound **3** in $CDCl_3$. *Denotes residual solvent peak

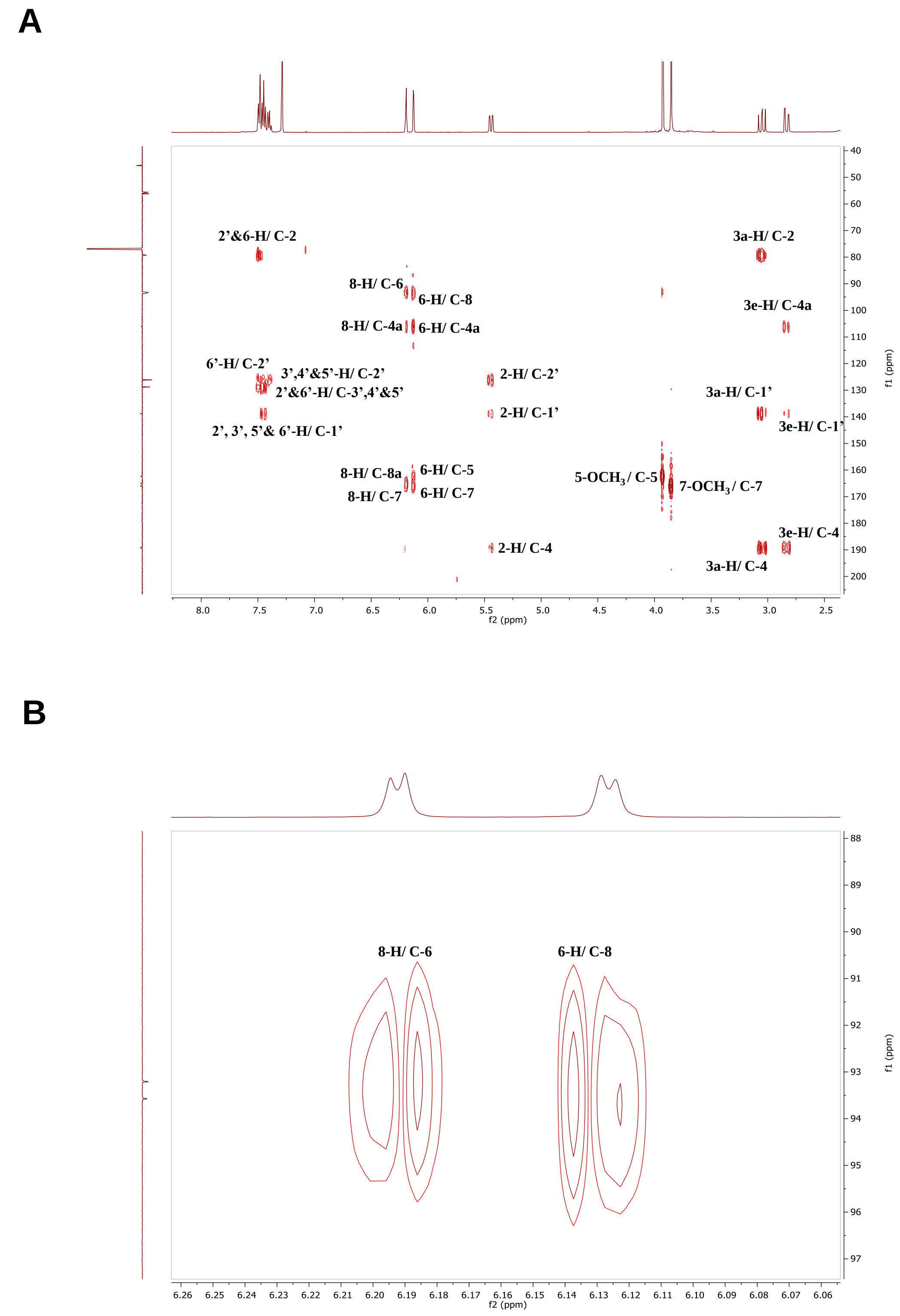

Supplementary material S17: (A) Full and (B) expansion of HMBC spectrum of compound **3** in $CDCl_3$.

A

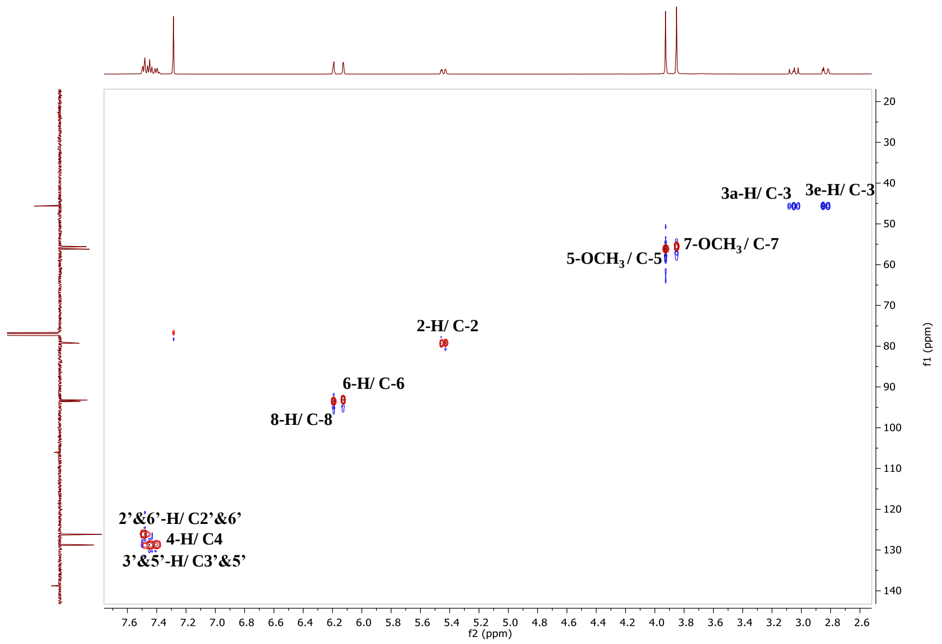

B

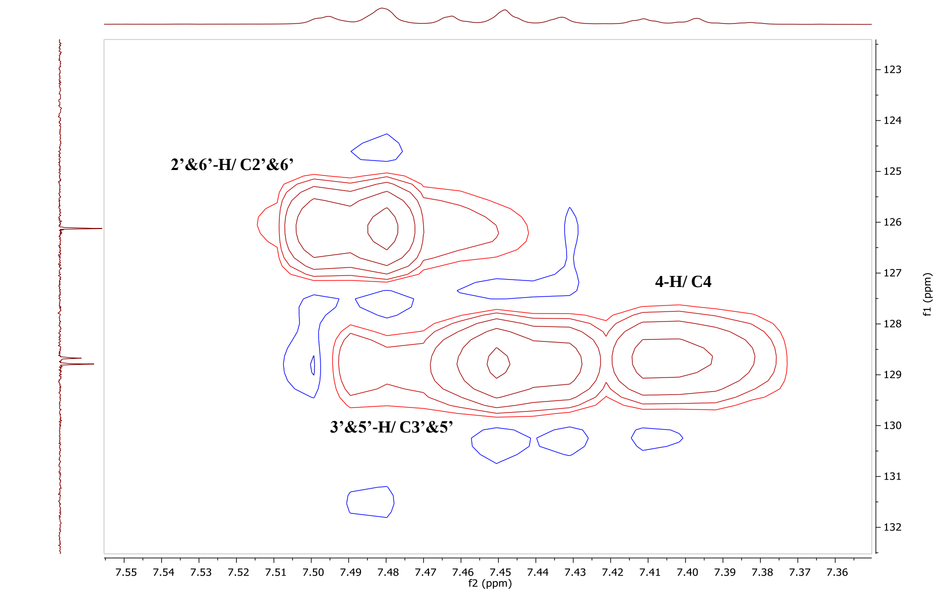

Supplementary material S18: (A) Full and (B) expansion of HSQC spectrum of compound **3** in $CDCl_3$.

Supplementary Table S3: NMR data for compound 3 in $CDCl_3$.

Position	δ_H (mult, *J* (Hz))	δ_C	HMBC
2	5.44 (1H, dd, 13.1, 2.8)	79.2	C-2′, C-1′, C-4
3	2.83 (1H, dd, 16.5, 2.8),<br>3.05 (1H, dd, 16.5, 13.1)	45.6	C-1′, C-4, C-10
4	-	189.2	-
5	-	162.3	-
6	6.13 (1H, d, 2.2)	93.2	C-8, C-5, C-7, C-10
7	-	166.0	-
8	6.19 (1H, d, 2.2)	93.6	C-6, C-9, C-10
9	-	165.0	-
10	-	106.0	-
1′	-	138.7	-
2′, 6′	7.5 (2H, m)	126.1	C-1′, C-2 , C-3′, C-4′, C-5′
3′, 4′, 5′	7.38 (3H, m)	128.7, 128.8	C-2′ , C-1′
5-OCH_3	3.98 (3H, s)	56.1	C-5
7-OCH_3	3.85 (3H , s)	55.6	C-7

Compound **4**

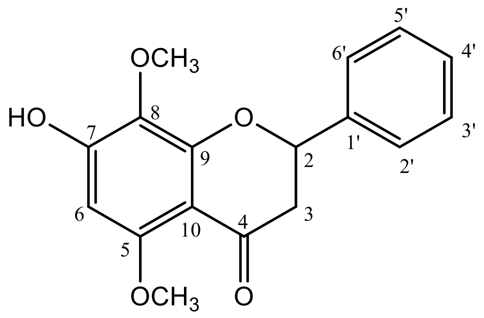

7-hydroxy-5,8–dimethoxy flavanone.

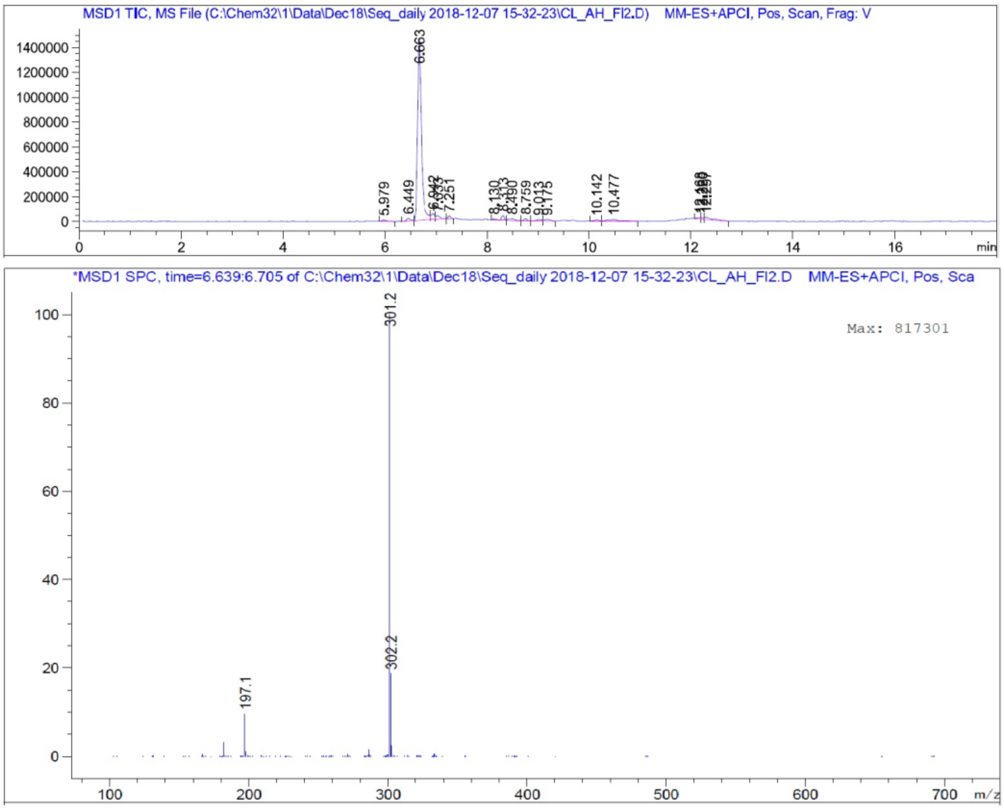

Supplementary material S19: LC-MS spectrum showing $[M+H]^+$ ion for compound **4**

A

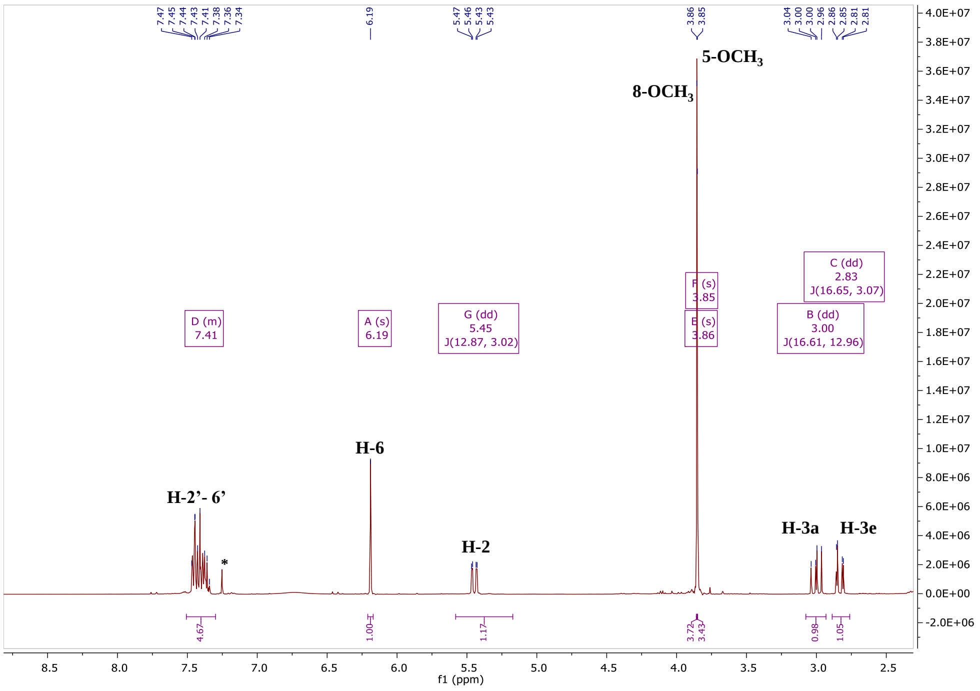

B

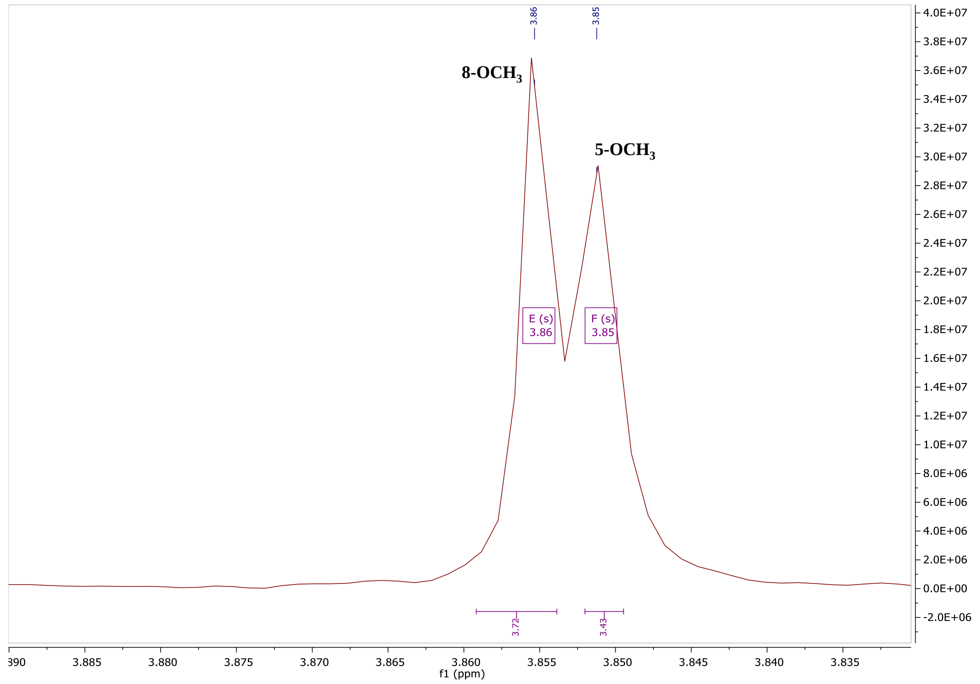

Supplementary material S20: (A) Full ^{1}H NMR spectrum (500 MHz) of compound **4** in $CDCl_3$ and (B) expansion of the methoxy region. *Denotes residual solvent peak.

A

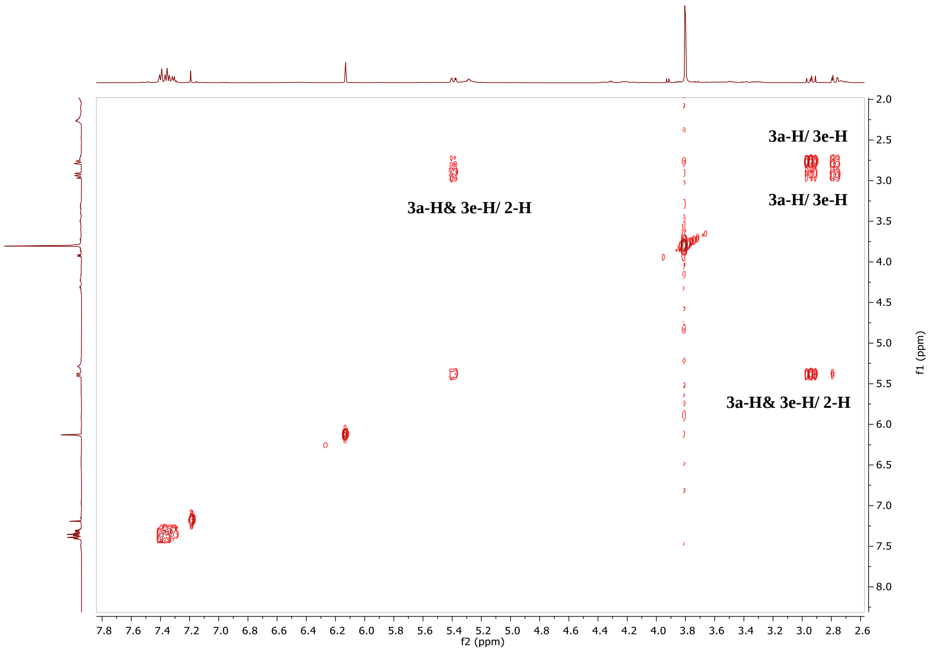

B

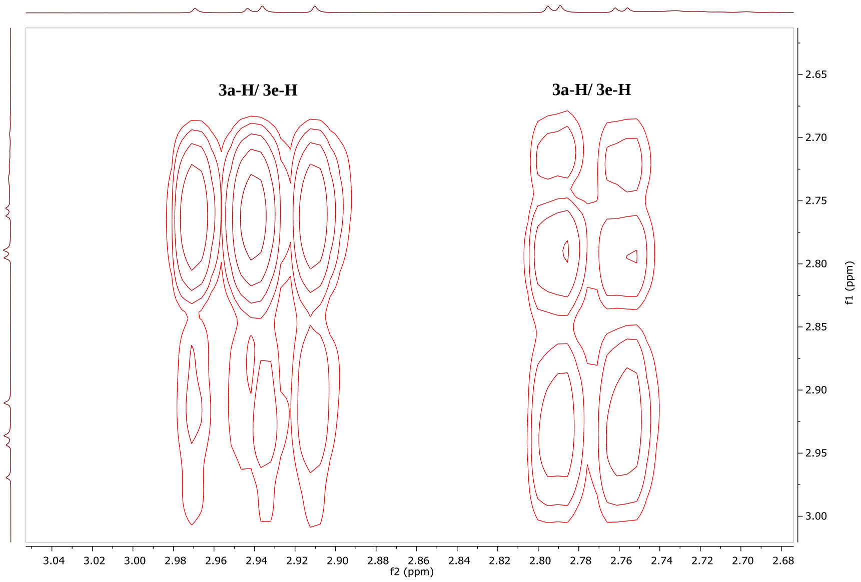

Supplementary Spectrum S21: (A) Full and (B) expansion of COSY spectrum of compound **4** in $CDCl_3$.

A

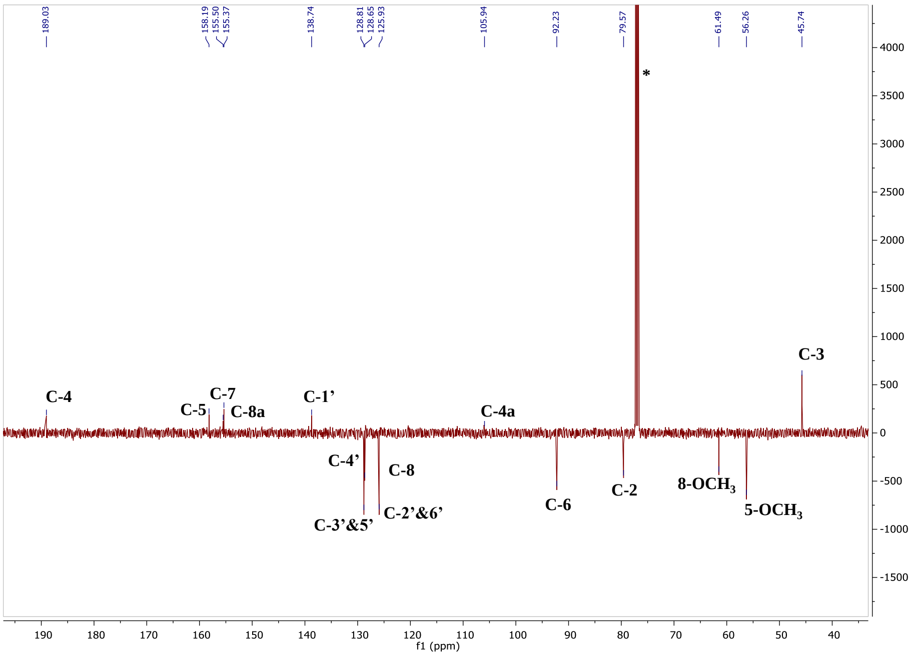

B

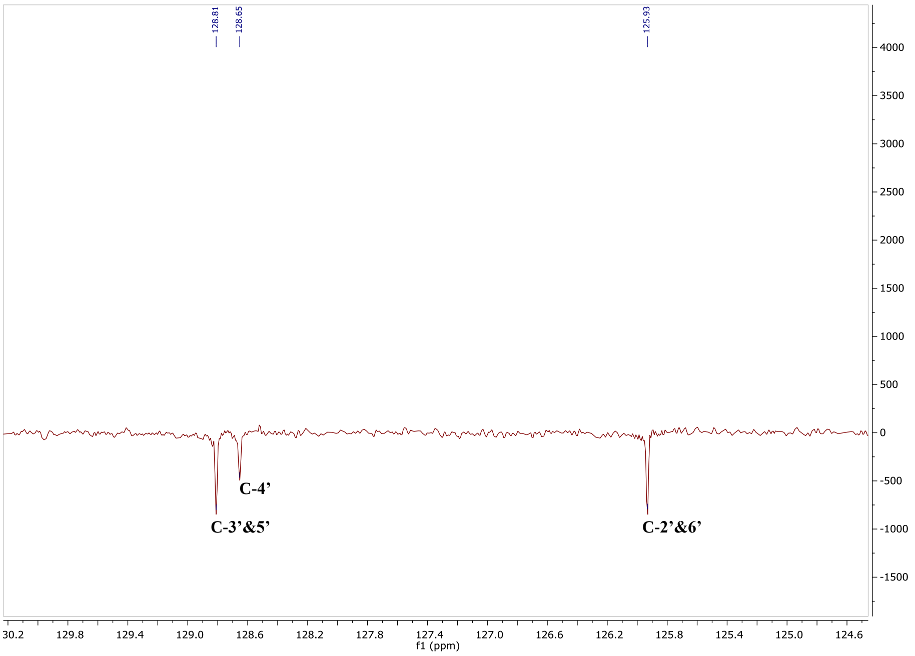

Supplementary material S22: (A) Full DEPTq 135 ^{13}C-NMR spectrum in $CDCl_3$ of compound **4** and (B) expansion of B ring signals.

A

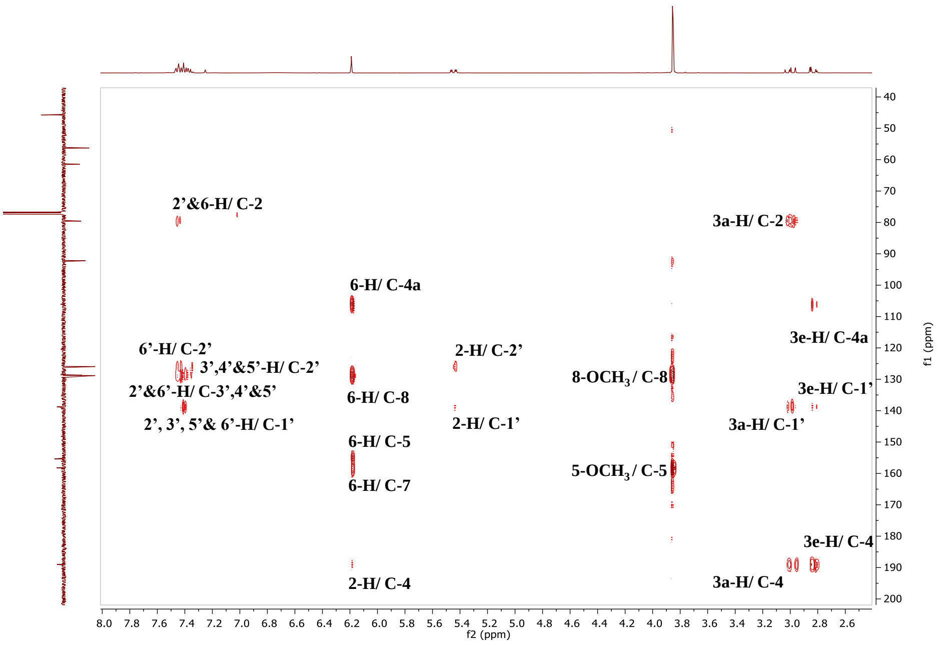

B

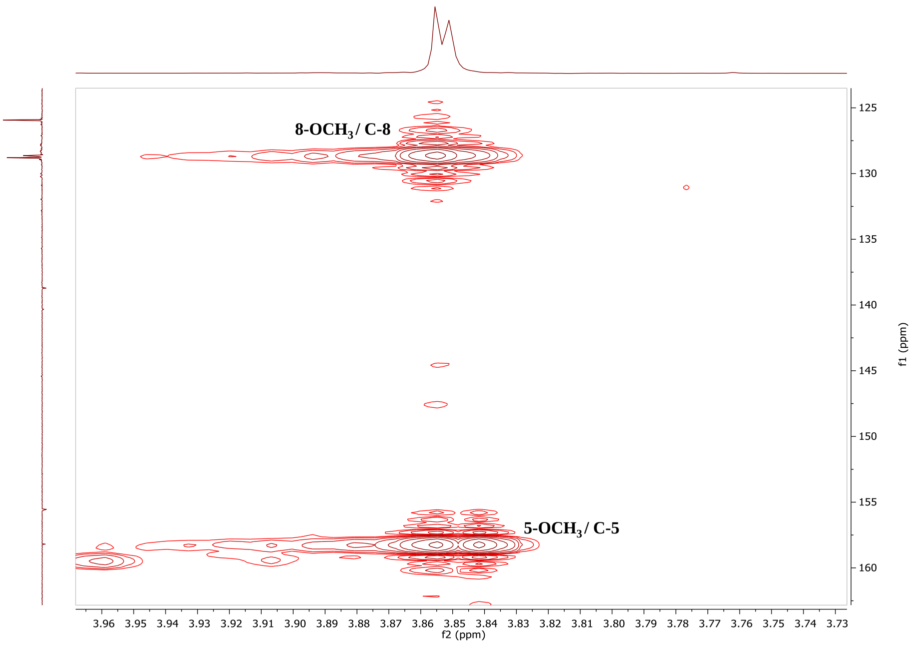

Supplementary material 23: (A) Full HMBC spectrum in $CDCl_3$ and (B) expansion of the methoxy region of compound **4**.

A

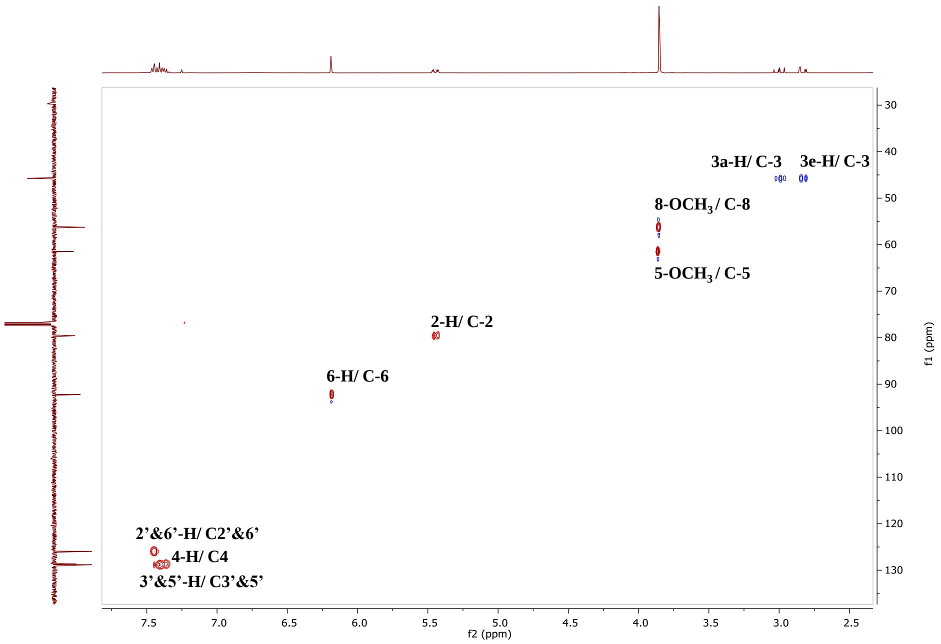

B

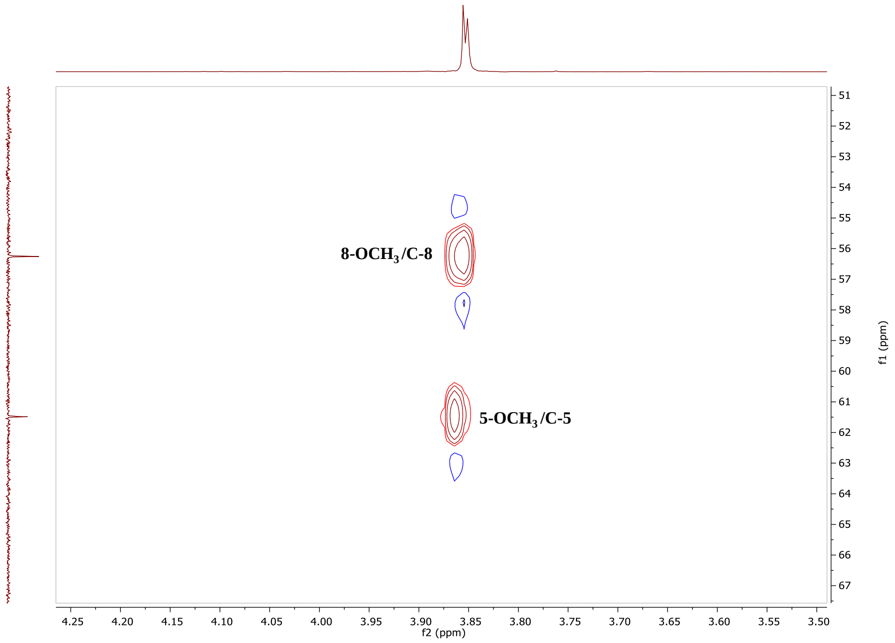

Supplementary material S24: (A) Full and (B) expansion of HSQC spectrum of compound **4** in $CDCl_3$.

Supplementary Table S4: NMR data for compound 4 in $CDCl_3$.

Position	δ_H (mult, *J* (Hz))	δ_C	HMBC
2	5.45 (1H, dd, 12.9, 3.0)	79.6	C-1′, C-2′, C-4
3	2.83 (1H, dd, 16.6, 3.1),<br>3.0 (1H, dd, 16.6, 13.0)	45.7	C-1′, C-4, C-10
4	-	189.0	-
5	-	158.1	-
6	6.20 (1H, s)	92.1	C-5, C-7, C-8, C-10
7	-	156.0	-
8	-	128.6	-
9	-	155.5	-
10	-	105.9	-
1′	-	138.7	-
2′, 6′	7.47 (2H, m)	125.9	C-2, C-3′, C-4′, C-5′
3′, 4′, 5′	7.34 (3H, m)	128.8	C-2′, C-1′
5-OCH_3	3.85 (3H, *s*)	56.1	C-5
8-OCH_3	3.86 (3H , *s*)	61.4	C-8
